# Characterization of the microbial communities and their correlations with volatile flavor compounds and physicochemical factors in Bashang suancai, a traditional Chinese pickle

**DOI:** 10.3389/fmicb.2024.1478207

**Published:** 2024-11-19

**Authors:** Yuan Liu, Chen Yin, Jian Wang, Weihai Xing, Yali Huang, Zhiyu Yan, Jiachen Chen, Yu Han, Weiran Zhu, Yidi Zhao, Kai Zhang, Tingting Tian, Xinru Guo, Lin Yuan, Yang Liu

**Affiliations:** ^1^Hebei Key Laboratory of Quality & Safety Analysis-Testing for Agro-Products and Food, Hebei North University, Zhangjiakou, China; ^2^College of Resources and Environmental Science, Hebei University of Science and Technology, Shijiazhuang, China; ^3^Zhangjiakou Food and Drug Inspection Center, Zhangjiakou, China

**Keywords:** Bashang suancai, microbial communities, volatile flavor compounds (VFCs), physicochemical characteristics, correlation analysis

## Abstract

Bashang suancai is one of the most wellknown traditional fermented vegetables in North China. The study examined the variations in bacterial diversity, physicochemical properties, and volatile flavor compounds (VFCs) of Bashang suancai over a 7-day fermentation period, utilizing Illumina NovaSeq sequencing and headspace solid phase microextraction-gas chromatography-mass spectrometry (HS-SPME-GC-MS). The leading bacterial phyla were *Firmicutes*, *Proteobacteria*, and *Cyanobacteria*, while the predominant bacterial species included *Vibrio*, *Lactiplantibacillus*, *Cyanobacteriales*, *Weissella*, and *Latilactobacillus*. The bacterial community diversity decreased significantly following 7 days of fermentation. The microbial profiles were markedly affected by pH, reducing sugar content (RSC), and salt content (SC). A total of 187 VFCs were identified from the specimens. Following 5 days of fermentation, the taste compounds achieved equilibrium, with isothiocyanates, alcohols, and esters predominating among the volatile molecules. Spearman correlation analysis revealed that a strong link between *Latilactobacillus*, *Levilactobacillus*, *Lactiplantibacillus*, *Weissella*, and *Vibrio* with the flavor of pickles. This study established a significant foundation for identifying strains that enhance taste development and improve the nutritional and sensory quality of Bashang suancai.

## Highlights

- The microbial community diversity decreased significantly after 7 days of fermentation.
- The markers of microbia and VFCs as well as their correlation were identified.
- The microbial profiles were significantly influenced by pH, RSC and SC.
- The flavor altered significantly in the first 5 days of fermentation.
- Three predominant species of lactobacillus were highly associated with 31 VFCs.

## 1 Introduction

Fermented vegetable pickles possess a rich history and considerable diversity in China, originating as early as the third century B.C. (Caplice and Fitzgerald, 1999). Suancai is a highly favored traditional pickle in China, particularly prevalent in northern regions due to its distinctive flavor and texture. Dongbei suancai, a famous exemplar of suancai, has garnered increasing interest from researchers in recent years. A multitude of studies has been conducted to establish effective guidelines for enhancing the fermentation process of suancai, focusing on isolation, screening, and inoculation of starter cultures (Zhao and Gu, 2019; Yang et al., 2023), the discovery of microbiota and associated flavors (Sun et al., 2023; Xiao et al., 2020), along with the impact of manufacturing process variables such as salt content, acidity, and temperature on fermentation (Liang et al., 2020; An et al., 2021; He et al., 2020). Nonetheless, the above researches predominantly overlooked the ecological factors influencing microbiota and flavor quality in spontaneously fermented vegetables, including raw materials (Nguyen et al., 2013; Park et al., 2019), geographical locations, and seasonal variations (Liang et al., 2018c; Liu et al., 2019).

Bashang suancai, as an essential part of the daily diet, is a popular traditional fermented vegetable throughout Bashang region (transition zone between Hebei Province and Mongolian Plateau), and its surrounding areas of Hebei, Inner Mongolia and Shanxi. The production process of Bashang suancai is similar to that of Dongbei suancai, but the types of vegetables used are indeed different. The raw material of Bashang suancai is common head cabbage (*Brassica oleracea* L. var. *capitata* L.), while the raw material of Dongbei suancai is Chinese cabbage (*Brassica rapa* L. ssp. *pekinesis*). Besides, Bashang suancai is generally fermented in summer or autumn, when high-altitude cold vegetables such as cabbage are ripe and harvested, while Dongbei suancai is fermented in winter or spring. What is more, Bashang area is a transition zone between grassland and plateau, which has a unique climate of large temperature difference between day and night, cool summer, drought and little rain, thus affecting the microflora balance in this area. Consequently, due to the diversity of raw materials, climatic circumstances, and geographical distribution (Jeong et al., 2013; Lee et al., 2017), Bashang suancai may possess a distinct microflora and metabolites, as well as unique flavor, taste, and texture. To our knowledge, there have been no research reported on the detection of bacterial communities and their relationships with the chemical profiles and physicochemical parameters of Bashang suancai. The regulation and optimization of the vegetable fermentation process, as well as the commercial development of genuine Bashang suancai products, have been significantly impacted. Therefore, it is imperative to comprehend the traditional microbial communities involved in the fermentation process and their specific contributions to the quality attributes of interest to consumers.

In recent years, high-throughput sequencing has proven to be an effective technique for analyzing the microbial community structure of fermented food (Lv et al., 2019; Zang et al., 2018; Zhang et al., 2018). Illumina NovaSeq 6000 sequencing is the most representative of the second generation sequencing technologies, which can obtain superior sequence quality and larger data volume than Miseq sequencing and Hiseq sequencing. In addition, the current volatile flavor compounds (VFCs) analysis in food mainly includes headspace solid phase microextraction-gas chromatography-mass spectrometry (HS-SPME-GC-MS), electronic nose (E-nose) and headspace-gas chromatography-ion mobility spectrometry (HS-GC-IMS) (Liu et al., 2023). HS-SPME-GC-MS is the most commonly used because of its convenience, high sensitivity and good reproducibility (Lerman et al., 2012).

Up to now, research on fermented vegetables mostly concentrates on the principal bacterial communities, volatile chemicals, and their interrelations post-fermentation, as well as the comparative analysis of these parameters among various pickles. However, there is a paucity of comprehensive studies examining the alterations in microbial diversity, taste compounds, and physicochemical properties of fermented vegetables over the fermentation period and their relationships. Additionally, the Bashang region possesses a distinctive environment characterized by significant diurnal temperature variation, chilly summers, aridity, and minimal precipitation, resulting in a unique microflora. Bashang suancai is a widely recognized traditional fermented vegetable in the Bashang region and its adjacent areas of Hebei, Inner Mongolia, and Shanxi. The impact of the distinct microbiota in the Bashang region on pickle fermentation has not been studied. The study provides broad insights on the field.

This study utilized cabbages (*Brassica oleracea* L. var. capitata L.) sourced from a local farm as raw materials for the production of Bashang suancai. The variations in microbial diversity, flavor compounds, physicochemical properties, and their interrelationships during the 7-day fermentation period were systematically investigated using high-throughput sequencing and chromatographic techniques, combined with principal component analysis (PCA), redundancy analysis (RDA), and Spearman correlation analysis. Our findings will lay the theoretical basis for the improvement of traditional fermentation process and further industrialized production of Bashang suancai.

## 2 Materials and methods

### 2.1 Bashang suancai preparation and sampling

Bashang suancai is prepared by pickling cabbage, white radish, and carrot, either individually or in combination, with cabbage being the predominant vegetable used. The Bashang suancai samples included in this study were home-made and naturally fermented in Kangbao County, Hebei Province, China, from June 19 to 26. The daily high temperature was 30°C, while the daily minimum temperature was 9°C. The production procedure was as follows: Fresh cabbages (*Brassica oleracea* L. var. capitata L.) sourced from a local cultivation site were stripped of outer leaves, rinsed, and sliced into square pieces of roughly 3 × 3 cm. The cabbage slices were thereafter placed in a pot, seasoned with 4% salt, and allowed to ferment spontaneously at an ambient temperature of 18–20°C for 7 days. A substantial weight was applied atop the cabbage to extract surplus moisture, thus preventing air from entering. During the fermentation process, brine and pickle samples were collected on days 0, 1, 3, 5, and 7, respectively, denoted as groups A, B, C, D and E. Three replicates were performed for each group sample. The brine samples were used for microbial communities analysis and the pickle samples were used for volatile compounds and physicochemical properties analysis. The samples were collected into 50 mL centrifuge tubes using sterile scoops, and immediately transported to the laboratory in a low temperature sampling box equipped with ice packs within 24 h for storage at −20°C.

### 2.2 Physicochemical properties analysis

The pH value of the sample was determined using a pH meter (PHS-3C, Leici, Shanghai, China). Total titratable acidity (TTA) was determined using a 1% phenolphthalein-ethanol solution as the indicator and titrating the brine with 0.1 mol/L NaOH until a reddish color was achieved. The salt content (SC) was measured utilizing a salt meter (Liang et al., 2018a). Nitrite content (NC) was determined according to the National food safety standard - Determination of nitrite and nitrate in food (GB 5009.33-2016, 2016) by the hydrochloric acid naphthyl ethylenediamine method. Reducing sugar content (RSC) was determined using direct titration according to the National food safety standard - Determination of reducing sugars in food (GB 5009.7-2016, 2016).

### 2.3 DNA extraction, PCR amplification and Illumina NovaSeq high-throughput sequencing

The metagenomic DNA from samples was extracted using the Rapid Bacterial Genomic DNA Isolation Kit (Sangon Biotech, Shanghai, China) according to the manufacturer’s instructions. The quality of the obtained DNA was determined by 1% agarose gel electrophoresis and spectrophotometry.

The V3–V4 regions of the bacteria 16S rRNA genes were amplified with the primer 338F (5′-ACTCCTACGGGAGGCAGCA-3′) and 806R (5′-GGACTACHVGGGTWTCTAAT-3′). The PCR programs were performed as follows: (1) predenaturation at 95°C for 5 min; (2) 20 cycles of denaturation at 95°C for 30 s, annealing at 50°C for 30 s and extension at 72°C for 40 s; (3) extension at 72°C for 7 min and storage at 4°C.

Amplification products from all samples were quantified using Quant-iT^™^ dsDNA HS Reagent (Thermo Fisher Scientific, MA, United States) and HS Buffer (Invitrogen, CA, United States), pooled in equimolar and pair-end sequenced using the Illumina NovaSeq 6000 platform (Beijing Biomarker Biotech Co., Ltd., China).

### 2.4 Bioinformatics analysis

Raw readings were filtered with Trimmomatic software (Version 0.33), and primers and barcodes were eliminated using cutadapt software (Version 1.9.1) to yield clean reads. Their integration was accomplished with FLASH software (Version 1.2.11). The assembly settings included a minimum overlap of 10 base pairs, a minimum similarity of 90% in the overlap region, and a maximum of 5 base pairs for mismatched bases. Effective readings were selected and potential chimeras were eliminated with UCHIME v4.2 software.

The aforementioned readings were clustered to create operational taxonomic units (OTUs) with Usearch software (version 10.0) at a 97% identity threshold (Edgar, 2013). Taxonomic classifications were assigned to amplicon sequence variants (ASVs) utilizing the Silva database (Version 138, https://www.arb-silva.de/) and employing the DADA2 methodology (Callahan et al., 2016) within QIIME2 software (Version 2020.6.0). Besides, the assessments of α-diversity and β-diversity were calculated using QIIME2 and visualized with R software (Version 2.15.3). Pearson’s correlation study, conducted with IBM SPSS 25.0 software, yielded the correlation indexes. Cytoscape (3.9.0) was utilized to illustrate the interaction networks between bacterial communities and flavor compounds (Saito et al., 2012).

### 2.5 Volatile flavor compounds analysis

The extraction and detection of VFCs in suancai samples were performed by headspace solid phase microextraction-gas chromatography-mass spectrometry (HS-SPME-GC-MS). Three grams of pickle samples were weighed, grinded, homogenized, and immediately transferred into a 20-mL headspace vial, and directly spiked with 10 μL 2-methyl-3-heptanone [5 μg/mL (w/v)] as the internal standard. The vial was incubated at 55°C for 20 min and the SPME fibers (DVB/CAR/PDMS, Supelco Inc.) were exposed to headspace at 55°C for 40 min for adsorption. Finally, the fibers were extracted and placed into a heated gas chromatography injection port for thermal desorption at 250°C for 3 min. The examination of volatile chemicals was performed using a Thermo Fisher GC-MS TRACE 1310/Q-Exactive Orbitrap (Thermo Fisher Scientific Inc., Waltham, MA, United States) fitted with a capillary column (VF-WAXms, 60 m × 0.25 mm i.d. × 0.25 μm film thickness). The oven temperature gradient was executed as outlined below: maintain 40°C for 2 min, then elevate to 230°C at a rate of 3°C/min and sustain for 5 min. The mass spectra were obtained at a source temperature of 230°C at 70 eV. The mass scan range of m/z was established from 30 to 400 amu, with an automatic-gain-control target value of 1E6. The identification and calculation parameters of volatile chemicals adhere to the protocol of Yang et al. (2022).

### 2.6 Statistical analysis

Significant differences were determined by one-way analysis of variance (ANOVA) and Duncan’s multiple range test using SPSS 22.0 software (SPSS Inc., Chicago, IL, United States). And the differences with *p*-values of <0.05 were defined as statistically significant. Data were presented as mean ± standard deviation. The graph presentations were generated by using the Origin 9.0 Software (OriginLab Corporation, Hampton, MA, United States). Three independent experiments were carried out for each treatment group.

## 3 Results and discussion

### 3.1 Analysis of physicochemical properties during fermentation

The changes of pH value, TTA, SC, NC and RSC during the fermentation of Bashang suancai are shown in Table 1. The pH showed an obvious decreasing trend during the fermentation, which was below 4 (3.34) on the 3rd day. Correspondingly, the TTA of suancai increased gradually, rising sharply from 1.82 to 9.09 g/L on day 3. It has been reported that the fermented cabbage is considered mature when the pH value is below 4.0 and TTA is greater than 3 g/L (Zhang et al., 2016). This suggested that the pickle was edible without the flavor of raw vegetables on the third day, aligning with the conclusions of Luo et al. (2021). Furthermore, during the late fermentation phase, the diminished pH level may be ascribed to the accumulation of organic acids resulting from microbial growth and metabolism (Wu et al., 2015).

**Table 1:** Changes of pH value, TTA, SC, NC and RSC during the fermentation of Bashang suancai.

Day	pH value	TTA (g/L)	SC (%)	NC (mg/kg)	RSC (g/100 g)
0	5.72 ± 0.17^a^	1.71 ± 0.04^d^	0.42 ± 0.02^d^	3.12 ± 0.07^e^	3.02 ± 0.17^a^
1	4.21 ± 0.26^b^	1.82 ± 0.05^d^	0.69 ± 0.05^c^	4.33 ± 0.13^d^	2.77 ± 0.09^b^
3	3.34 ± 0.02^c^	9.09 ± 0.06^c^	0.98 ± 0.06^b^	5.56 ± 0.14^c^	2.55 ± 0.08^bc^
5	3.22 ± 0.17^d^	9.99 ± 0.09^a^	1.03 ± 0.09^ab^	17.92 ± 0.31^a^	2.32 ± 0.11^cd^
7	3.21 ± 0.26^d^	9.54 ± 0.09^b^	1.12 ± 0.07^a^	11.04 ± 0.30^b^	2.29 ± 0.12^d^

Note: Values with superscript letters a, b, c, and d are significantly different across columns (*p* < 0.05).

Consequently, the concentration of sugar, being the favored carbon source for microbial growth, diminished significantly during the fermentation process. As fermentation time increased, the SC exhibited a moderate upward trend, maintaining a low range of 0.42–1.12%. The NC peaked (17.92 ± 0.31 mg/kg) on the 5th day, but subsequently decreased, and the daily NC was lower than the safety limit (20 mg/kg) in accordance with the National Food Safety Standard-Limits of Contaminants in Food (GB 2762-2017). The reduction of NC after 5 days of fermentation may be ascribed to diminished nitrate reductase activity resulting from elevated acidity (An et al., 2020). During fermentation, the RSC decreased rapidly due to microbial carbohydrate metabolism, while the concentration of organic acids rose.

### 3.2 Volatile flavor compounds analysis of Bashang suancai during fermentation

The flavor profile of Bashang suancai was examined over a 7-day fermentation period utilizing the HS-SPME-GC-MS technology. A total of 187 volatile flavor compounds (VFCs) were identified in all suancai samples, comprising 4 hydrocarbons, 3 terpenes, 25 alcohols, 22 aldehydes, 13 ketones, 41 esters, 6 acids, 5 furans, 12 sulfur compounds, 11 nitrogen compounds, 5 sulfur-nitrogen compounds, 8 phenols, 4 benzene ethers, 12 benzene hydrocarbons, and 16 other compounds (Supplementary Table S1).

**Figure 1:**
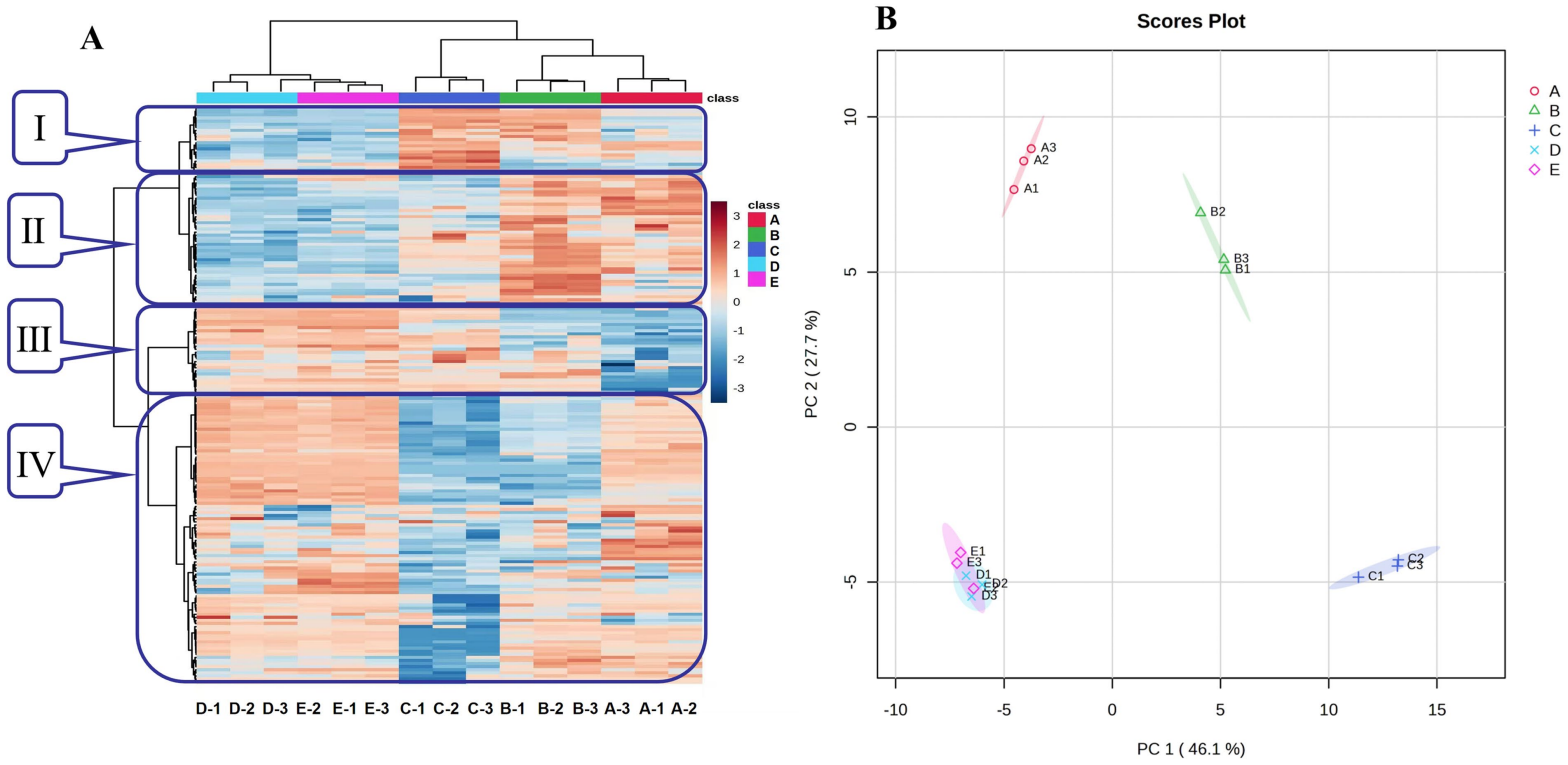
Heatmap analysis (A) and PCA plot (B) of VFCs during fermentation of Bashang suancai. A, B, C, D and E represented samples collected on 0 day, 1 day, 3 days, 5 days, and 7 days, respectively.

**Figure 2:**
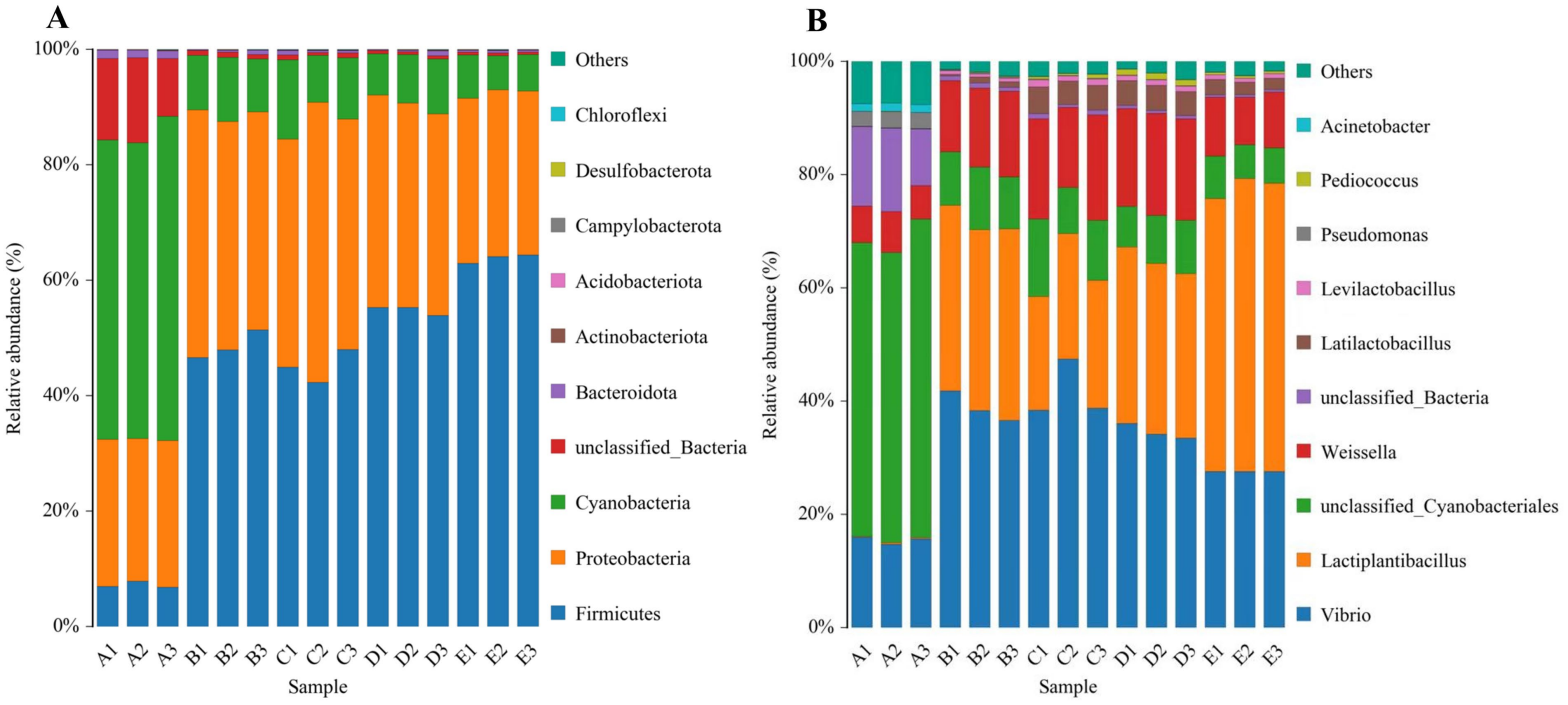
The relative abundance of microbial communities during fermentation of Bashang suancai. The bacterial community at the phylum level during fermentation (A), and the bacterial community at the genus level during fermentation (B). A, B, C, D and E on the horizontal coordinate represented samples collected on 0 day, 1 day, 3 days, 5 days, and 7 days, respectively. Three replicates were performed for each suancai sample. The phyla and genera with the top 10 relative abundance are presented, respectively.

**Figure 3:**
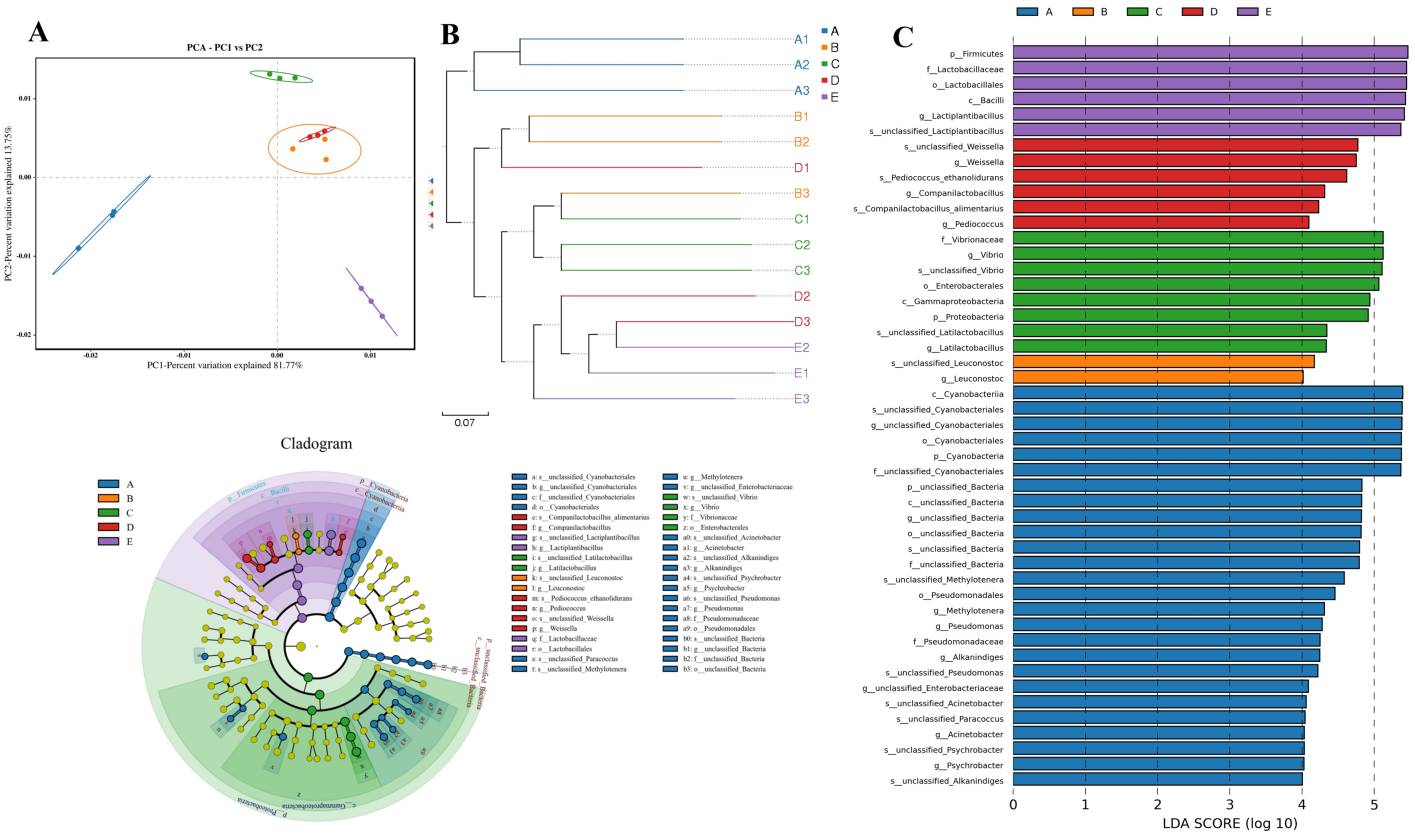
Comparison of β-diversity indices of bacterial communities and redundancy analysis (RDA) of microorganisms and internal environmental factors during fermentation of Bashang suancai. (A) UniFrac (unweighted) principal component analysis (PCA) scores plot according to bacterial diversity. (B) Multiple samples UPGMA clustering tree. (C) LEfSe analyses of different bacteria taxa in Bashang suancai during 7 days of fermentation. Histogram of the results of the microbiota of Bashang suancai with a threshold value of 4. *p*-values <0.05 considered significant. Labels beginning with o-indicate order; f-family; g-genus; s-species. Cladogram representing the abundance of those taxa in Bashang suancai during the different stages of fermentation. A, B, C D and E represented samples collected on 0 day, 1 day, 3 days, 5 days, and 7 days, respectively. Three replicates were performed for each suancai sample.

The heat map analysis and PCA were used to compare the differences and similarities of the VFCs among Bashang suancai samples throughout the entire fermentation process (Figure 1). As shown in Figure 1A, the compounds in zone I initially increased, peaked on day 3, and subsequently declined; the compounds in zone II were minimal during the early fermentation phase but gradually rose, whereas the compounds in zone III exhibited an almost inverse trend; the compounds in zone IV demonstrated a pattern of initial decline followed by an increase, attaining their lowest level on the third day. Overall, the flavor compounds on the third day exhibited the most significant difference from other groups, and the chemicals appeared to stabilize after 5 days of fermentation. The PCA results (Figure 1B) indicated a clear differentiation of variations in VFCs among the samples. The cumulative variance contribution of PC1 (46.1%) and PC2 (27.7%) was 73.8%, signifying sufficient sample information. Samples from groups A, B, and C occupied distinct quadrants, however samples from groups D and E were located in the same quadrant and in proximity to one another, suggesting that a clear differentiation between the two groups was not achieved. The results indicated that the flavor of suancai underwent substantial changes from the beginning until the fifth day of fermentation, after which the flavor composition began to stabilize. This was consistent with the findings of the heat map study (Figure 1A). However, the PCA results of bacterial communities (Figure 2A) indicated a significant disparity in bacterial composition between groups D and E. It was reasonable to infer that the variance in microbial diversity had minimal impact on the flavor of the pickle at the conclusion of fermentation. To further investigate and validate the differences in flavor compounds among the groups, an orthogonal partial least squares discriminant analysis (OPLS-DA) model was developed to assess the variety of the compounds (Figure 3). The results were in accordance with those of the PCA analysis.

**Figure 4:**
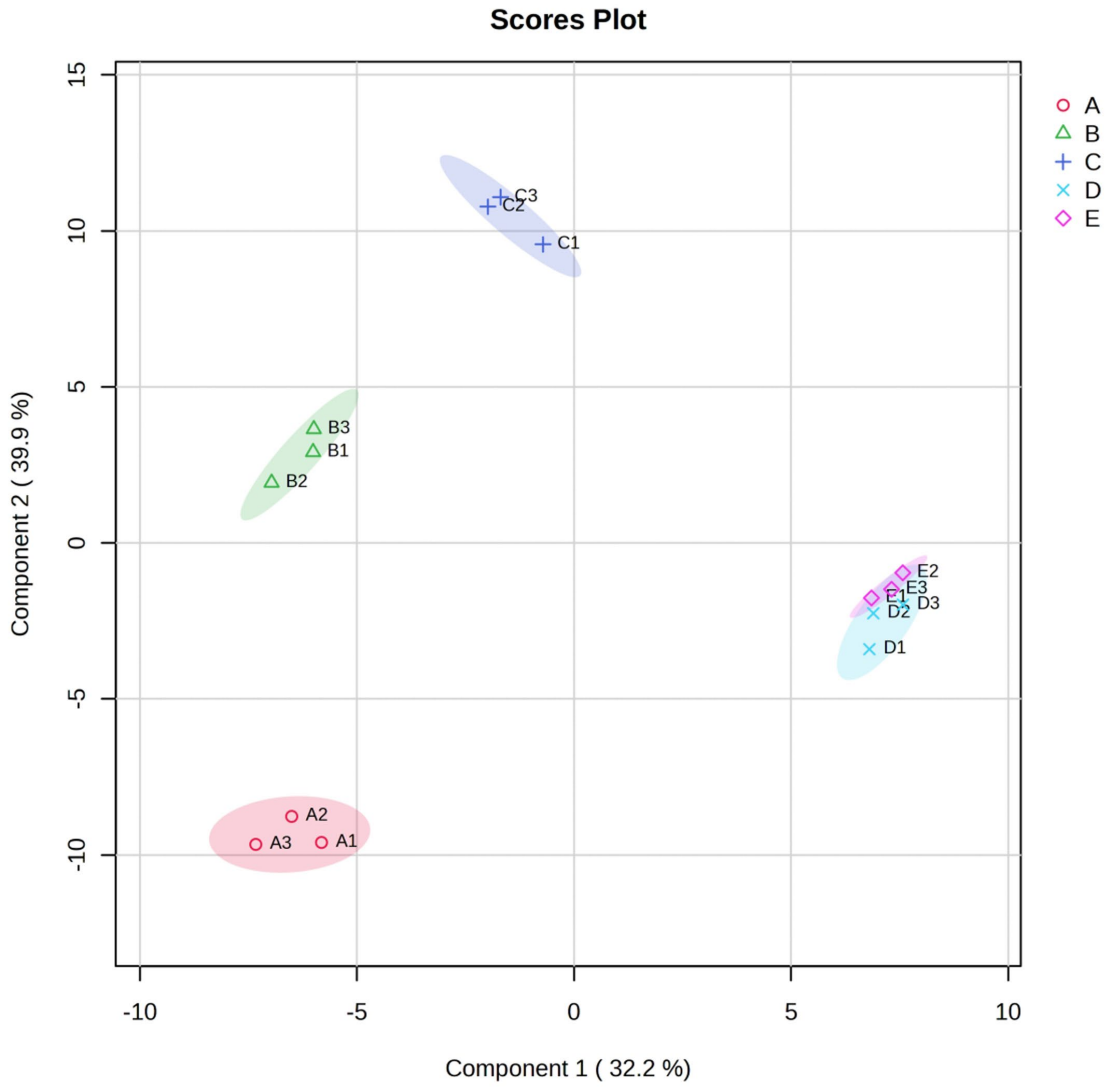
The scatter plot based on PLS modeling was performed in VFCs of Bashang suancai samples during various fermentation stages. Different colors and shapes indicated different fermentation times, with red triangle, green cross, blue fork, light blue rhombus and purple inverted triangle indicating the 0, 1st, 3rd, 5th and 7th day of fermentation, respectively. A, B, C, D and E represented samples collected on 0 day, 1 day, 3 days, 5 days, and 7 days, respectively. Three replicates were performed for each suancai sample.

The variations in VFCs of Bashang suancai throughout fermentation are illustrated in Figure 4A and Supplementary Table S2. On the 7th day of fermentation, sulfur-nitrogen compounds (38.19%), alcohols (33.79%), and esters (6.28%) predominated among the 17 types of aromatic compounds. Allyl isothiocyanate and 4-Isothiocyanato-1-butene were the most significant sulfur-nitrogen compounds, supporting the conclusions of Liang et al. (2020). The isothiocyanates identified in suancai samples primarily originate from the cabbage utilized in fermentation, serving as breakdown products of glucosinolates, which are prevalent plant secondary metabolites (Nugrahedi et al., 2017). The volatile chemicals attained their maximum and minimum values on the 3rd and 5th days of fermentation, imparting distinctive aromas to cabbage and other cruciferous vegetables (Zhou et al., 2018). Alcohols are regarded as another significant sensory components in fermented foods, contributing scents of pepper, citrus, rose, cheese, and sweet fruit (Cagno et al., 2016). The alcohols, such as benzeneethanol, 3-methyl-1-butanol, 2-nonanol, and 1-hexanol, exhibited a pattern of initial increase followed by a decline, likely attributable to the accumulation of heterozygosity in lactic acid bacteria and yeast metabolism. The elevated concentration of alcohols facilitated ester formation and aided in regulating the rise of sulfide content (Luo et al., 2021). Esters were the third most significant flavor compounds in fermented products, characterized by sweet or fruity aromas, which enhance the flavor of fermented meals by alleviating unpleasant odors (Lee et al., 2013). As fermentation progressed, the concentration of esters (e.g., hexadecanoic acid ethyl ester, formic acid octyl ester, and methyl phenylacetate) exhibited an initial increase followed by a decline.

The research conducted by Li et al. (2022) indicated that the ultimate quality of pickles was affected by the type and concentration of organic acids, which are chiefly accountable for the sour flavor. Figure 4A illustrats that the organic acids, including acetic acid, hexanoic acid, and octanoic acid, first decreased steadily but surged dramatically at the end. In the early stage of fermentation, it may be the overflow of water in vegetables reduced the concentration of acid, while in the late stage of fermentation, the accumulation of metabolites such as carbohydrates, amino acids and nucleotides increased the concentration of acid (Fernández and Zúñiga, 2006; Gutsche et al., 2012), which was fundamentally caused by the growth of *Lactobacillus* capable of producing lactic acid (Wu et al., 2015).

Aldehydes and ketones were known to greatly enhance the flavor of fermented products due to their low odor thresholds (Zhao et al., 2022). The findings indicated the presence of nonanal, benzaldehyde, benzeneacetaldehyde, and heptanal in samples exhibiting rose, citrus, nutty, and sweet herbaceous scents. Ketones mostly included acetoin, 3-octanone, and 3-nonen-2-one, which were identified as products of lipid and amino acid degradation during microbial fermentation (Devanthi and Gkatzionis, 2019).

In order to deeply investigate the particular differential VFCs across different fermentation stages, the variable importance in projection (VIP) plot was employed to encapsulate the significance of the variables. Figure 4B illustrats that 46 chemicals (VIP >1.0) were identified as substantially correlated with the fermentation process. Moreover, the VIP values of methyl phenylacetate, isoamyl lactate, ethyl 2-methylbutyrate, 5-methylhexanenitrile, cis-rose oxide, heptyl acetate, 2H-pyran-2-one, tetrahydro-6-propyl- and 2-heptyl-furan were above 2.0, indicating their significance as volatile chemicals during fermentation. Therefore, these flavor substances have become the key factors to be considered in the following correlation analysis.

### 3.3 Composition analysis of bacterial community during fermentation

A total of 1,031,016 effective bacterial reads were collected from 15 samples by Illumina NovaSeq sequencing analysis, with a valid sequence length of 424.47 bp. Supplementary Table S3 illustrates the alpha diversities of the microbial community during the fermentation of Bashang suancai. The overall count of operational taxonomic units (OTUs) at 97% sequence identity was substantial across all samples, ranging from 205 to 404. The Chao1 and ACE indices indicated bacterial richness, but the Shannon and Simpson indices represented bacterial community diversity, which varied among the brine samples. After 7 days of fermentation, the diversity of bacterial composition in the brine samples diminished, suggesting that the bacterial community became homogeneous at the end of the fermentation process. Besides, the coverage rate per sample exceeded 99%, indicating that the sequencing depth was adequate to elucidate the true composition of the microbial communities throughout the fermentation process.

**Figure 5:**
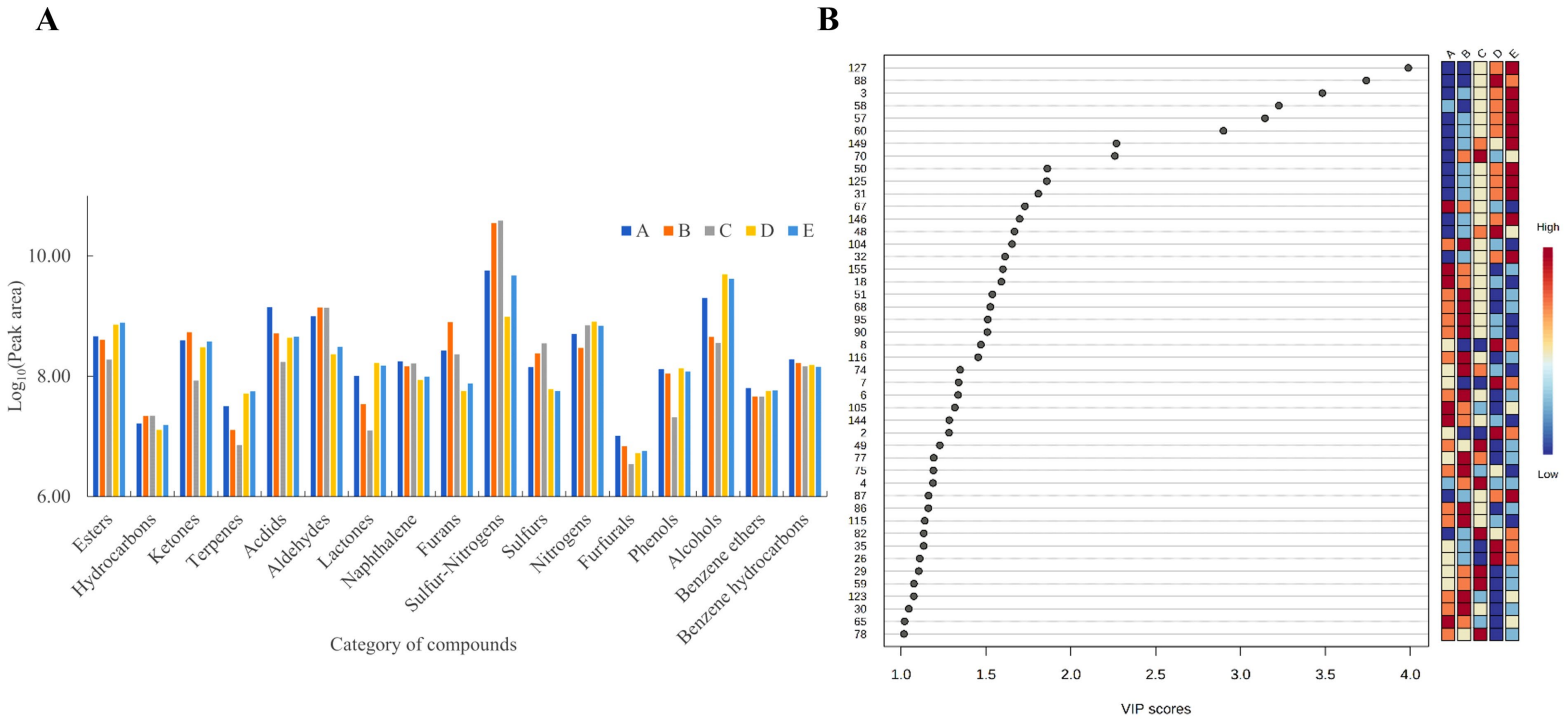
Changes (A) and VIP plot (B) of VFCs during fermentation of Bashang suancai. A, B, C, D and E represented samples collected on 0 day, 1 day, 3 days, 5 days, and 7 days, respectively.

The statistical analysis revealed that the community structure of all samples encompassed 25 phyla, 48 classes, 114 orders, 225 families, 411 genera, and 474 species. Figure 5A delineates the 10 phyla with the greatest abundance within each fermentation time category. *Firmicutes*, *Proteobacteria*, and *Cyanobacteria* were identified as the predominant bacterial phyla during fermentation, consistent with prior research on Dongbei suancai (He et al., 2020; Sun et al., 2023) in Northeast China. The significant prevalence of *Cyanobacteria* distinguished Bashang suancai in our research from other pickles like Sichuan paocai, potentially linked to contamination from specific raw materials, preparation techniques, or inadequate sanitary conditions during manufacturing (Xiao et al., 2020). *Firmicutes* (6.76–64.35%) grew from the initial day of fermentation and dominated the entire fermentation process, whereas *Proteobacteria* (24.72–48.52%) experienced an initial increase followed by a decline, although the fluctuations were not significant. In contrast, *Cyanobacteria* (5.94–56.25%) showed a marked decline from the onset of fermentation, maintaining a low abundance (5.94–13.71%) during the 1 to 7-day fermentation period.

At the genus level, the main genera, including *Vibrio*, *Lactiplantibacillus*, *Cyanobacteriales*, *Weissella*, *Latilactobacillus*, etc. (Figure 5B), were detected with a high abundance at different stages of fermentation. Among them, *Lactiplantibacillus* increased rapidly and was observed as the dominant genus (48.19–51.77%) on the 7th day. Besides, *Weissella* and *Latilactobacillus* also showed a significant increase during fermentation. These findings were consistent with previous research indicating that *Lactobacillus* was the predominant genus in Chinese fermented vegetables (Liu et al., 2019; Liang et al., 2018b). However, the *Vibrio* and *Cyanobacteriales* discovered in this research have rarely been reported in similar fermented foods. Their existence was likely associated with inadequate sanitary conditions, which might readily lead to contamination during the fermentation process. Moreover, a notable disparity in low abundance genera was observed between the Bashang suancai studied and the Sichuan paocai derived from several vegetable species, featuring prevalent bacteria such as *Enterobacter* and *unclassified Enterobacteriaceae* (Cagno et al., 2016). This likely indicated the intrinsic heterogeneity in the spontaneous fermentation of vegetables from diverse origins. According to Rao et al. (2020), the development of various microbial communities in each pickle variety mostly relied on the specific vegetable species, together with ambient or processing conditions. During the initial phases of fermentation, the population of *Cyanobacteriales* diminished significantly by 40.22 to 47.09%, perhaps due to the ability of *lactobacilli* to convert sugars into acids and generate antibacterial compounds that suppressed the proliferation of rival bacteria (Suwannaphan, 2021).

### 3.4 Comparison of the bacterial communities in different fermentation stages of suancai

PCA was a prevalent method for dimensionality reduction classification that analyzed variations in bacterial community structure during the fermentation process using unweighted UniFrac distances, as illustrated in Figure 2A. PC1 and PC2 represented 81.77 and 13.75% of the cumulative percentage variance of species, respectively, demonstrating that adequate sample information was supplied (Liu et al., 2021). The distribution map distinctly delineated several fermentation stages within reasonably autonomous areas. The results indicated that Group A was clearly separated from the other groups, suggesting that the bacterial composition prior to fermentation was markedly different from that following fermentation. The data distributions of groups B and D were clustered, indicating that the microbial communities of suancai were analogous on the 1st and 5th days of fermentation. Conversely, the samples from groups C and E were positioned far at the intersection of the top left and upper right quadrants and the bottom right quadrant, respectively. The results indicated a apparent discrepancy presented in bacterial compositions between the 3rd and 7th days of fermentation compared to other phases of fermentation.

The cluster-tree (unweighted UniFrac) was utilized to compare the bacterial community structures in samples with varying fermentation durations. As illustrated in Figure 2B, all samples were categorized into two clusters. Clusters B, C, D, and E indicated similarity among the bacterial communities throughout fermentation, but group A formed a distinct cluster, demonstrating considerable differences. Moreover, groups C and E had the least number of tiny branches among the fermentation stage groups, indicating substantial variation in their bacterial compositions. The above results were consistent with the PCA analysis findings in our study.

Linear discriminant analysis Effect Size (LEfSe) was employed to compare the bacterial communities during the fermentation process of Bashang suancai across varying fermentation durations (Figure 2C). The genera *Cyanobacteriales*, *Acinetobacter*, *Alkanindiges*, *Psychrobacter*, *Pseudomonas* were the microbial biomarkers in group A. *Leuconostoc* was the microbial biomarker in group B. *Latilactobacillus* and *Vibrio* were the biomarkers in group C. *Companilactobacillus*, *Pedioccoccus* and *Weissella* were the biomarkers in group D, while *Lactiplantibacillus* was the biomarker in group E.

### 3.5 Correlation between microbial community and physicochemical properties during fermentation

**Figure 6:**
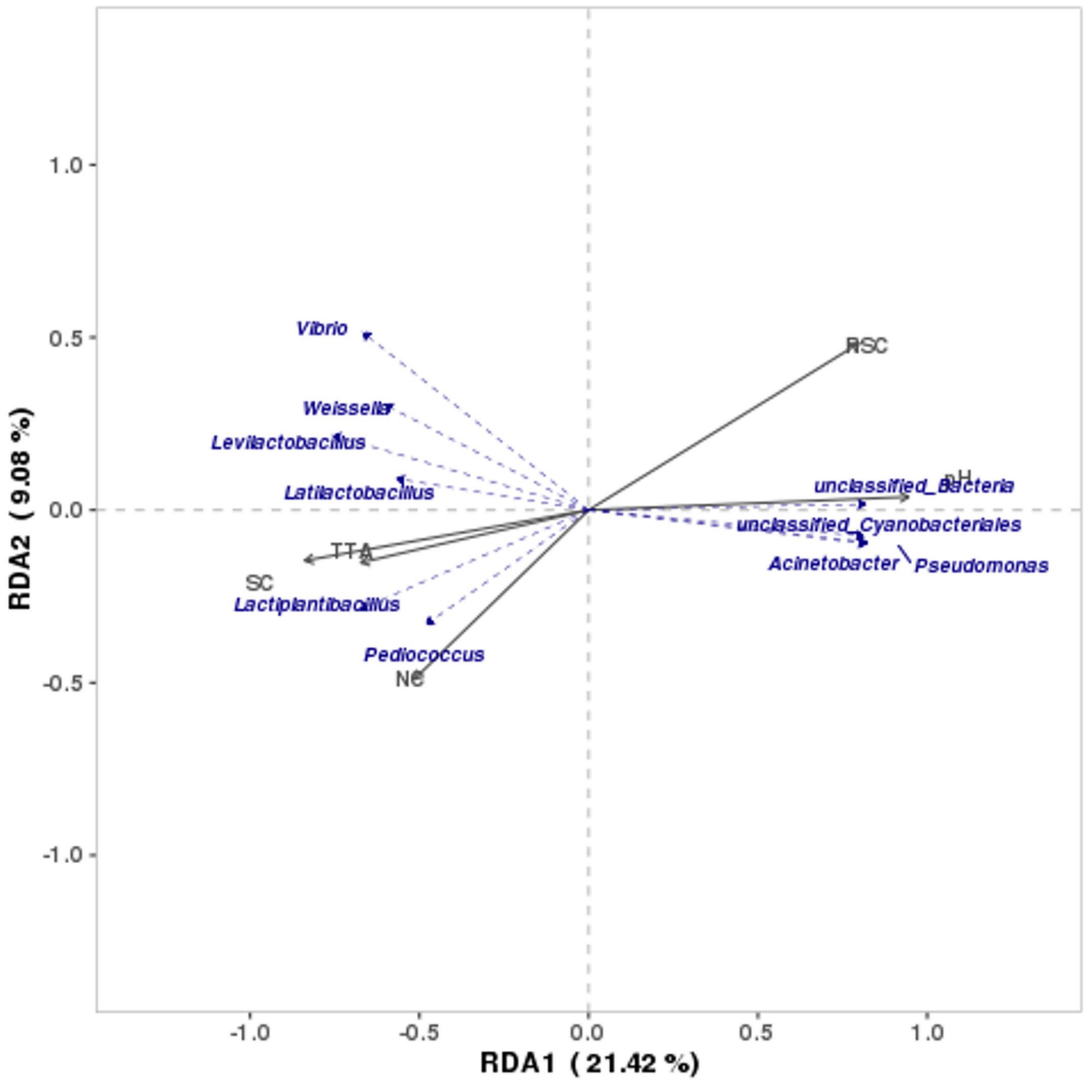
RDA analysis of bacterial community structure and physicochemical quality indexes. The lengths of the curved arrows represent the strength of the environmental factor’s influence on community change, and the directions of them indicate the routes of data points on the score plots during fermentation.

In the study, RDA was conducted to outline the relationship between microbial community and internal environmental factors of Bashang suancai. Figure 6 illustrates that the first two canonical axes accounted for 9.08 and 21.42% of the variation, respectively. The RDA results indicated that pH predominantly influenced the microbial populations, followed by RSC and SC. The pH and RSC exhibited a negative correlation with SC, TTA, and NC. The inverse relationship between pH and NC contradicted the findings of He et al. (2020) and Liang et al. (2020). This may result from the gradual development of NC during the initial 5 days of fermentation, followed by a fall in this study, in contrast to the NC in the above two reports, which rapidly reached a peak post-fermentation before subsequently decreasing. The potential rationale may be attributed to the lower salinity seen in this study compared to the above two studies. Xiong et al. (2016) showed that salinity significantly affected the initial phase of spontaneous fermentation. Yang et al. (2019) discovered that elevated salt levels could diminish nitrite accumulation, potentially due to the suppression of nitrite-producing microbial development (Liang et al., 2020). Furthermore, our research revealed that salt exerted a considerable positive effect on TTA and a significant negative effect on pH values during fermentation. Similar findings have also been documented by Yang et al. (2022) and Zhao et al. (2022).

*Lactiplantibacillus*, *Latilactobacillus*, *Levilactobacillus*, and *Weissella*, as predominant genera in late fermentation, exhibited a strong positive correlation with SC and TTA (*p* < 0.05) and a significant negative correlation with pH (*p* < 0.05). This aligned with the prior findings indicating that *Lactobacillus* and *Weissella* proliferated at elevated salt concentrations (Lee et al., 2017). The lactic acid bacteria not only lower the pH value but also create an anaerobic environment favorable for their proliferation (Moon et al., 2018). Conversely, a large amount of genera prevalent in the initial phase of fermentation, including *unclassified Cyanobacteriales*, *unclassified Bacteria*, *Acinetobacter*, and *Pseudoalteromonas*, were significantly positively correlated with pH (*p* < 0.05). The bacteria adhering to the raw material often originated from the environment and initiated the fermentation process (Xiao et al., 2018). No microorganisms exhibited a significant correlation with salinity and glucose in this study, which was consistent with the results of He et al. (2020).

### 3.6 Correlation between microbiota and flavor compounds in Bashang suancai during fermentation

**Figure 7:**
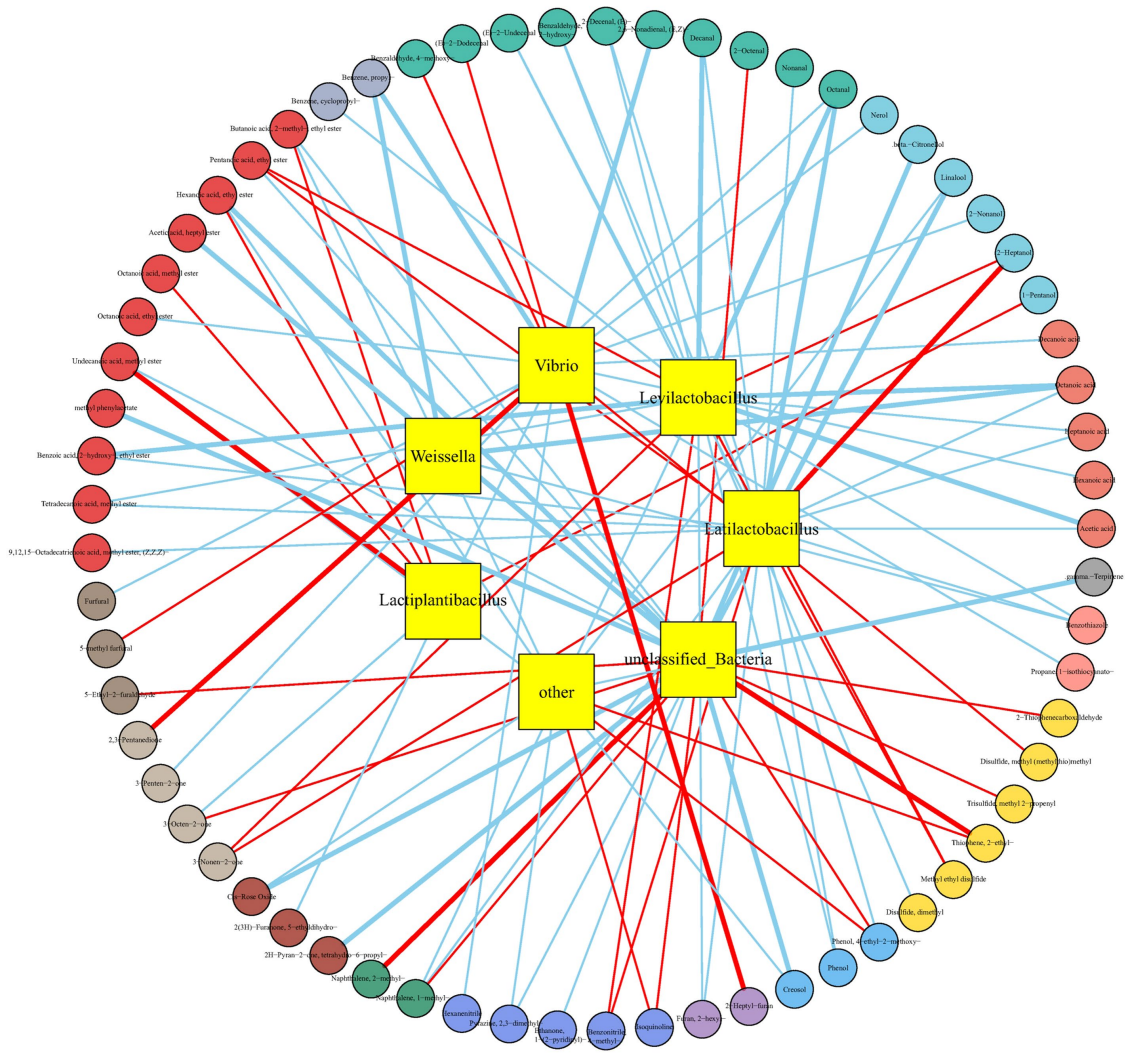
Correlation networks between microbial taxa at the genus level and flavor compounds in Bashang suancai samples during fermentation. The inner circles indicate microbial genus, while the outer circles indicate chemical compounds. The absolute value of the Pearson rank correlation coefficient is >0.7. The red lines connecting the circles represent positive correlations, while the blue lines represent negative correlations. The thickness of the line indicates the magnitude of the correlation. The thicker the line, the stronger the correlation. The correlation coefficients and *p*-values are shown in Supplementary Table S4.

To investigate the roles of microorganisms in the formation of VFCs in Bashang suancai samples, the Spearman’s rank correlation between seven dominant bacterial genera and volatile compounds was conducted and shown in Figure 7 and Supplementary Table S4. *Latilactobacillus*, *Levilactobacillus*, and *Lactiplantibacillus* exhibited a significant positive correlation (*r* > 0.70) with the production of alcohols, acids, esters, ketones, benzonitriles, and sulfides, while demonstrating a significant negative correlation (*r* < −0.70) with the formation of organic acids, aldehydes, naphthalenes, furans, phenols, and benzothiazoles. The three *Lactobacillus* species were closely associated to 31 flavor compounds. Consequently, they were likely to be recognized as the primary flavour-contributing microbiota of the pickle, similar to the findings described by Xiao et al. (2020). They found that *Lactobacillus acetotolerans* and *Lactobacillus sakei* may be considered marker functional bacteria of the three typical traditional Chinese fermented vegetable foods: Jiangxi yancai, Sichuan paocai and Dongbei suancai. Besides, Stankus (2014) and Han et al. (2021) indicated that *Weissella* genera exhibited substantial positive correlations with the production of diverse volatile compounds during the fermentation of doenjang and pickles. However, no remarkable positive correlation between *Weissella* and flavor formation was found in this study. Nevertheless, it was observed to be highly negatively correlated (*r* < −0.70) with some odorous compounds such as octanoic acid, octanal, propyl benzene and benzothiazole. The findings underscored the distinctive function of *Weissella* in the chemical compositions of Bashang suancai samples, which was quite distinct from previous studies. This may be due to the variation in *Weissella* species (e.g., *Weissella cibaria*) levels among different fermented foods. Furthermore, the environmental conditions during the fermentation process, such as temperature, salinity, and the types of vegetables, etc., also exert a discernible impact. Consequently, a more comprehensive exploration and rigorous analysis are imperative to gain deeper insights. *Vibrio* was another predominant genus in Bashang suancai, with a relative abundance reaching 27.56% on the 7th day. It was a prevalent genus in domestic pickles in Northeast China (An et al., 2021) and in homemade Chaozhou Sauerkraut (Huang et al., 2023). Due to the relatively open production environment and non-standardized process conditions of household manual pickles based on spontaneous fermentation, apart from beneficial bacteria such as *Lactobacillus*, it is easy to introduce a small number of harmful microorganisms such as *Vibrio* or spoilage bacteria into the final product through the raw materials and processing environment. These genera can not only deteriorate the flavor of fermented vegetables, but also provide possible food safety risks because to their pathogenicity (Chien et al., 2023; Karruli et al., 2023).

## 4 Conclusion

In this study, the bacterial diversities, physicochemical properties and volatile compounds of Bashang suancai samples across a 7-day fermentation period were systematically analyzed for the first time. The primary phyla observed were *Firmicutes*, *Proteobacteria*, and *Cyanobacteria*, whereas the leading genera during fermentation included *Vibrio*, *Lactiplantibacillus*, *Cyanobacteriales*, *Weissella*, and *Latilactobacillus*. The *Vibrio* and *Cyanobacteriales* identified in our investigation were rarely found in previous similar fermented foods, perhaps due to the raw materials and spontaneous fermentation methods employed. The disparity in microbiota was particularly pronounced on days 3 and 7 of fermentation. The RDA results indicated that the microbial profiles were significantly influenced by pH, RSC, and SC.

A total of 187 volatile flavor compounds were discovered from Bashang suancai samples utilizing the HS-SPME-GC-MS method. The flavor profiles of suancai altered significantly in the first 5 days of fermentation, subsequently stabilizing. At the end of fermentation, isothiocyanates, alcohols, and esters predominated among the 17 aromatic compounds. Spearman correlation analysis indicated that *Latilactobacillus*, *Levilactobacillus*, and *Lactiplantibacillus* were found highly associated with 31 flavor compounds in Bashang suancai samples, whereas *Weissella* predominantly suppressed the development of specific undesirable odors, including octanoic acid, octanal, propyl benzene, and benzothiazole. These results may facilitate the manufacture of industrial-grade local characteristic pickles with healthful and safe quality, and unique flavor.

## Data Availability

The original contributions presented in the study are publicly available. This data can be found here: https://www.ncbi.nlm.nih.gov/sra/PRJNA1185291.

## References

[ref1] An F. Li M. Zhao Y. Zhang Y. Mu D. Hu X.. (2020). Metatranscriptome-based investigation of flavor-producing core microbiota in different fermentation stages of dajiang, a traditional fermented soybean paste of Northeast China. Food Chem. 343:128509. doi: 10.1016/j.foodchem.2020.128509, PMID: 33199116

[ref2] An F. Sun H. Wu J. Zhao C. Li T. Huang H.. (2021). Investigating the core microbiota and its influencing factors in traditional Chinese pickles. Food Res. Int. 147:110543. doi: 10.1016/j.foodres.2021.110543, PMID: 34399520

[ref3] Cagno R. D. Filannino P. Gobbetti M. (2016). “Fermented foods: fermented vegetables and other products” in Encyclopedia of food and health. eds. Finglas P. M. Toldrá F. (Oxford: Academic Press), 668–674.

[ref4] Callahan B. J. McMurdie P. J. Rosen M. J. Han A. W. Johnson A. J. A. Holmes S. P. (2016). DADA2: high-resolution sample inference from Illumina amplicon data. Nat. Methods 13, 581–583. doi: 10.1038/nmeth.3869, PMID: 27214047 PMC4927377

[ref5] Caplice E. Fitzgerald G. F. (1999). Food fermentations: role of microorganisms in food production and preservation. Int. J. Food Microbiol. 50, 131–149. doi: 10.1016/S0168-1605(99)00082-3 10488849

[ref6] Chien H. I. Yen Y. F. Lee Y. C. Wei P. C. Huang C. Y. Tseng C. H.. (2023). Determination of the bacterial community of mustard pickle products and their microbial and chemical qualities. Biology 12:258. doi: 10.3390/biology12020258, PMID: 36829535 PMC9953598

[ref7] Devanthi P. V. P. Gkatzionis K. (2019). Soy sauce fermentation: microorganisms, aroma formation, and process modification. Food Res. Int. 120, 364–374. doi: 10.1016/j.foodres.2019.03.010, PMID: 31000250

[ref8] Edgar R. C. (2013). UPARSE: highly accurate OTU sequences from microbial amplicon reads. Nat. Methods 10, 996–998. doi: 10.1038/nmeth.2604 23955772

[ref9] Fernández M. Zúñiga M. (2006). Amino acid catabolic pathways of lactic acid bacteria. Crit. Rev. Microbiol. 32, 155–183. doi: 10.1080/10408410600880643, PMID: 16893752

[ref10] Gutsche K. A. Tran T. B. T. Vogel R. F. (2012). Production of volatile compounds by *Lactobacillus sakei* from branched chain α-keto acids. Food Microbiol. 29, 224–228. doi: 10.1016/j.fm.2011.06.010, PMID: 22202876

[ref11] Han D. M. Chun B. H. Kim H. M. Jeon C. O. (2021). Characterization and correlation of microbial communities and metabolite and volatile compounds in doenjang fermentation. Food Res. Int. 148:110645. doi: 10.1016/j.foodres.2021.110645, PMID: 34507720

[ref12] He Z. Chen H. Wang X. Lin X. Ji C. Li S.. (2020). Effects of different temperatures on bacterial diversity and volatile flavor compounds during the fermentation of suancai, a traditional fermented vegetable food from northeastern China. LWT 118:108773. doi: 10.1016/j.lwt.2019.108773

[ref13] Huang W. Peng H. Chen J. Yan X. Zhang Y. (2023). Bacterial diversity analysis of Chaozhou sauerkraut based on high-throughput sequencing of different production methods. Fermentation 9:282. doi: 10.3390/fermentation9030282

[ref14] Jeong S. H. Lee H. J. Jung J. Y. Lee S. H. Seo H. Y. Park W. S.. (2013). Effects of red pepper powder on microbial communities and metabolites during kimchi fermentation. Int. J. Food Microbiol. 160, 252–259. doi: 10.1016/j.ijfoodmicro.2012.10.015, PMID: 23290232

[ref15] Karruli A. Catalini C. D’Amore C. Foglia F. Mari F. Harxhi A.. (2023). Evidence-based treatment of *Pseudomonas aeruginosa* infections: a critical reappraisal. Antibiotics 12:399. doi: 10.3390/antibiotics12020399, PMID: 36830309 PMC9952410

[ref16] Lee K. E. Lee S. M. Choi Y. H. Hurh B. S. Kim Y. S. (2013). Comparative volatile profiles in soy sauce according to inoculated microorganisms. Biosci. Biotechnol. Biochem. 77, 2192–2200. doi: 10.1271/bbb.130362, PMID: 24200796

[ref17] Lee M. Song J. H. Jung M. Y. Lee S. H. Chang J. Y. (2017). Large-scale targeted metagenomics analysis of bacterial ecological changes in 88 kimchi samples during fermentation. Food Microbiol. 66, 173–183. doi: 10.1016/j.fm.2017.05.002, PMID: 28576366

[ref18] Lerman N. L. D. Bellincontro A. Mencaelli F. Moreno J. Peinado R. A. (2012). Use of electronic nose, validated by GC-MS, to establish the optimum off-vine dehydration time of wine grapes. Food Chem. 130, 447–452. doi: 10.1016/j.foodchem.2011.07.058

[ref19] Li X. Cheng X. Yang J. Wang X. Lu X. (2022). Unraveling the difference in physicochemical properties, sensory, and volatile profiles of dry chili sauce and traditional fresh dry chili sauce fermented by *Lactobacillus plantarum* PC8 using electronic nose and HS-SPME-GC-MS. Food Biosci. 50:102057. doi: 10.1016/j.fbio.2022.102057

[ref20] Liang H. Chen H. Ji C. Lin X. Zhang W. Li L. (2018a). Dynamic and functional characteristics of predominant species in industrial paocai as revealed by combined DGGE and metagenomic sequencing. Front. Microbiol. 9:2416. doi: 10.3389/fmicb.2018.02416, PMID: 30356774 PMC6189446

[ref21] Liang H. Chen H. Zhang W. Yu C. Ji C. Lin X. (2018b). Investigation on microbial diversity of industrial Zhacai paocai during fermentation using high-throughput sequencing and their functional characterization. LWT 91, 460–466. doi: 10.1016/j.lwt.2018.01.088

[ref22] Liang H. He Z. Wang X. Song G. Chen H. Lin X.. (2020). Effects of salt concentration on microbial diversity and volatile compounds during suancai fermentation. Food Microbiol. 91:103537. doi: 10.1016/j.fm.2020.103537, PMID: 32539973

[ref23] Liang H. Yin L. Zhang Y. Chang C. Zhang W. (2018c). Dynamics and diversity of a microbial community during the fermentation of industrialized Qingcai paocai, a traditional Chinese fermented vegetable food, as assessed by Illumina MiSeq sequencing, DGGE and qPCR assay. Ann. Microbiol. 68, 111–122. doi: 10.1007/s13213-017-1321-z

[ref24] Liu Z. Peng Z. Huang T. Xiao Y. Li J. Xie M.. (2019). Comparison of bacterial diversity in traditionally homemade paocai and Chinese spicy cabbage. Food Microbiol. 83, 141–149. doi: 10.1016/j.fm.2019.02.012, PMID: 31202405

[ref25] Liu Q. Wu H. J. Luo J. Liu J. W. Zhao S. Q. Hu Q. H.. (2021). Effect of dielectric barrier discharge cold plasma treatments on flavor fingerprints of brown rice. Food Chem. 352:129402. doi: 10.1016/j.foodchem.2021.129402 33690074

[ref26] Liu Y. Zhang X. Wang J. Xing W. Liu Y. Wan Y.. (2023). Characterization of the volatile compounds in white radishes under different organic fertilizer treatments by HS-GC-IMS with PCA. Flavour Fragr. J. 38, 83–94. doi: 10.1002/ffj.3726

[ref27] Luo F. Yang Z. Zhong K. Huang C. Yu Z. Peng Z.. (2021). Effects of *Bacillus megaterium* L222 on quality and bacterial diversity of Sichuan paocai. Food Res. Int. 140:109994. doi: 10.1016/j.foodres.2020.109994, PMID: 33648228

[ref28] Lv J. Yang Z. Xu W. Li S. Liang H. Ji C.. (2019). Relationships between bacterial community and metabolites of sour meat at different temperature during the fermentation. Int. J. Food Microbiol. 307:108286. doi: 10.1016/j.ijfoodmicro.2019.108286, PMID: 31400632

[ref29] Moon S. H. Kim C. R. Chang H. C. (2018). Heterofermentative lactic acid bacteria as a starter culture to control kimchi fermentation. LWT 88, 181–188. doi: 10.1016/j.lwt.2017.10.009

[ref30] Nguyen D. T. L. Van Hoorde K. Cnockaert M. De Brandt E. Aerts M. Binh Thanh L.. (2013). A description of the lactic acid bacteria microbiota associated with the production of traditional fermented vegetables in Vietnam. Int. J. Food Microbiol. 163, 19–27. doi: 10.1016/j.ijfoodmicro.2013.01.024, PMID: 23500611

[ref31] Nugrahedi P. Y. Dekker M. Verkerk R. (2017). “Processing and preparation of *Brassica* vegetables and the fate of glucosinolates” in Glucosinolates, reference series in phytochemistry. eds. Mérillon J. M. Ramawat K. (Cham: Springer), 407–429.

[ref32] Park S. E. Seo S. H. Kim E. J. Byun S. H. Na C. S. Son H. S. (2019). Changes of microbial community and metabolite in kimchi inoculated with different microbial community starters. Food Chem. 274, 558–565. doi: 10.1016/j.foodchem.2018.09.032, PMID: 30372979

[ref33] Rao Y. Qian Y. Tao Y. She X. Li Y. Chen X.. (2020). Characterization of the microbial communities and their correlations with chemical profiles in assorted vegetable Sichuan pickles. Food Control 113:107174. doi: 10.1016/j.foodcont.2020.107174

[ref34] Saito R. Smoot M. E. Ono K. Ruscheinski J. Wang P. L. Lotia S.. (2012). A travel guide to Cytoscape plugins. Nat. Methods 9, 1069–1076. doi: 10.1038/nmeth.2212 23132118 PMC3649846

[ref35] Stankus T. (2014). Pickled vegetable condiments: a global industry and its literature. J. Agric. Food Inf. 15, 3–18. doi: 10.1080/10496505.2013.858048

[ref36] Sun X. H. Qi X. Han Y. D. Guo Z. J. Cui C. B. Lin C. Q. (2023). Characteristics of changes in volatile organic compounds and microbial communities during the storage of pickles. Food Chem. 409:135285. doi: 10.1016/j.foodchem.2022.135285, PMID: 36586248

[ref37] Suwannaphan S. (2021). Isolation, identification and potential probiotic characterization of lactic acid bacteria from Thai traditional fermented food. AIMS Microbiol. 7, 431–446. doi: 10.3934/microbiol.2021026, PMID: 35071941 PMC8712534

[ref38] Wu R. Yu M. Liu X. Meng L. Wang Q. Xue Y.. (2015). Changes in flavour and microbial diversity during natural fermentation of suan-cai, a traditional food made in Northeast China. Int. J. Food Microbiol. 211, 23–31. doi: 10.1016/j.ijfoodmicro.2015.06.028, PMID: 26159472

[ref39] Xiao M. Huang T. Huang C. Hardie J. Peng Z. Xie M.. (2020). The microbial communities and flavour compounds of Jiangxi yancai, Sichuan paocai and Dongbei suancai: three major types of traditional Chinese fermented vegetables. LWT 121:108865. doi: 10.1016/j.lwt.2019.108865

[ref40] Xiao Y. Xiong T. Peng Z. Liu C. G. Huang T. Yu H.. (2018). Correlation between microbiota and flavours in fermentation of Chinese Sichuan paocai. Food Res. Int. 114, 123–132. doi: 10.1016/j.foodres.2018.06.051, PMID: 30361008

[ref41] Xiong T. Li J. Liang F. Wang Y. Guan Q. Xie M. (2016). Effects of salt concentration on Chinese sauerkraut fermentation. LWT 69, 169–174. doi: 10.1016/j.lwt.2015.12.057

[ref42] Yang X. Hu W. Jiang A. Xiu Z. Ji Y. Guan Y.. (2019). Effect of salt concentration on quality of Chinese northeast sauerkraut fermented by *Leuconostoc mesenteroides* and *Lactobacillus plantarum*. Food Biosci. 30:100421. doi: 10.1016/j.fbio.2019.100421

[ref43] Yang M. Lai H. Wang Y. Mei Y. Huang Y. Zeng X.. (2023). Characterizing the impact of species/strain-specific *Lactiplantibacillus plantarum* with community assembly and metabolic regulation in pickled Suancai. Food Res. Int. 174:113650. doi: 10.1016/j.foodres.2023.113650, PMID: 37986488

[ref44] Yang Y. Li J. Xing J. Xing W. Tang C. Rao Z.. (2022). Untargeted profiling and differentiation of volatiles in varieties of meat using GC Orbitrap MS. Foods 11:3997. doi: 10.3390/foods11243997, PMID: 36553738 PMC9777611

[ref45] Zang J. Xu Y. S. Xia W. S. Yu D. W. Gao P. Jiang Q. X.. (2018). Dynamics and diversity of microbial community succession during fermentation of Suan yu, a Chinese traditional fermented fish, determined by high throughput sequencing. Food Res. Int. 111, 565–573. doi: 10.1016/j.foodres.2018.05.076, PMID: 30007719

[ref46] Zhang Q. S. Chen G. Shen W. X. Wang Y. Zhang W. X. Chi Y. L. (2016). Microbial safety and sensory quality of instant low-salt Chinese paocai. Food Control 59, 575–580. doi: 10.1016/j.foodcont.2015.06.041

[ref47] Zhang J. Wu S. S. Zhao L. H. Ma Q. L. Li X. Ni M. Y.. (2018). Culture-dependent and-independent analysis of bacterial community structure in Jiangshui, a traditional Chinese fermented vegetable food. LWT 96, 244–250. doi: 10.1016/j.lwt.2018.05.038

[ref48] Zhao W. Gu C. (2019). *Lactobacillus hulanensis* sp. nov., isolated from suancai, a traditional Chinese pickle. Int. J. Syst. Evol. Microbiol. 69, 2147–2152. doi: 10.1099/ijsem.0.003453, PMID: 31120413

[ref49] Zhao C. Tian Z. Yi J. Shi Y. Zhu J. Ji Z.. (2022). Characterization and correlation of bacterial community and volatile flavor compounds in Xiguajiang, a Chinese traditional fermented condiment. Food Res. Int. 162:111904. doi: 10.1016/j.foodres.2022.111904, PMID: 36461178

[ref50] Zhou Q. Tang H. Jia X. Zheng C. Huang F. Zhang M. (2018). Distribution of lucosinolate and pungent odors in rapeseed oils from raw and microwaved seeds. Int. J. Food Prop. 21, 2296–2308. doi: 10.1080/10942912.2018.1514632

