# Supplementary material for: Characterization of the microbial communities and their correlations with volatile flavor compounds and physicochemical factors in Bashang suancai, a traditional Chinese pickle
Source: Front Microbiol. 2024 Nov 19;15:1478207. doi: 10.3389/fmicb.2024.1478207 (PMC11613424; doi:10.3389/fmicb.2024.1478207)
Supplement: Supplementary file 1 [file Table_1.docx]

**Table S1.** HS-SPME-GC-MS integration parameters of VFCs in Bashang suancai samples. Rt: retention time, RI: retention index.

| No. | Classification | Component Name | CAS No. | Formula | Rt | RI |
| --- | --- | --- | --- | --- | --- | --- |
| 1 | Esters | Butanoic acid, ethyl ester | 105-54-4 | C6H12O2 | 10.682 | 1027 |
| 3 |  | Butanoic acid, 2-methyl-, ethyl ester | 7452-79-1 | C7H14O2 | 11.139 | 1042 |
| 8 |  | 1-Butanol, 3-methyl-, acetate | 123-92-2 | C7H14O2 | 13.285 | 1111 |
| 11 |  | Pentanoic acid, ethyl ester | 539-82-2 | C7H14O2 | 13.645 | 1122 |
| 19 |  | Acetic acid, pentyl ester | 628-63-7 | C7H14O2 | 15.038 | 1162 |
| 22 |  | Hexanoic acid, methyl ester | 106-70-7 | C7H14O2 | 15.452 | 1175 |
| 31 |  | Hexanoic acid, ethyl ester | 123-66-0 | C8H16O2 | 17.077 | 1222 |
| 37 |  | Acetic acid, hexyl ester | 142-92-7 | C8H16O2 | 18.502 | 1262 |
| 40 |  | Heptanoic acid, methyl ester | 106-73-0 | C8H16O2 | 18.974 | 1277 |
| 47 |  | 3-Hexen-1-ol, acetate, (Z)- | 3681-71-8 | C8H14O2 | 20.025 | 1307 |
| 53 |  | Heptanoic acid, ethyl ester | 106-30-9 | C9H18O2 | 20.586 | 1323 |
| 60 |  | Acetic acid, heptyl ester | 112-06-1 | C9H18O2 | 21.955 | 1363 |
| 64 |  | Octanoic acid, methyl ester | 111-11-5 | C9H18O2 | 22.52 | 1380 |
| 69 |  | Octanoic acid, ethyl ester | 106-32-1 | C10H20O2 | 24.066 | 1426 |
| 73 |  | Formic acid, octyl ester | 112-32-3 | C9H18O2 | 24.553 | 1442 |
| 79 |  | Nonanoic acid, methyl ester | 1731-84-6 | C10H20O2 | 25.928 | 1484 |
| 85 |  | Nonanoic acid, ethyl ester | 123-29-5 | C11H22O2 | 27.303 | 1527 |
| 88 |  | Isoamyl lactate | 19329-89-6 | C8H16O3 | 28.226 | 1557 |
| 93 |  | Decanoic acid, methyl ester | 110-42-9 | C11H22O2 | 29.141 | 1586 |
| 102 |  | Decanoic acid, ethyl ester | 110-38-3 | C12H24O2 | 30.427 | 1629 |
| 103 |  | Citronellyl acetate | 150-84-5 | C12H22O2 | 30.45 | 1630 |
| 107 |  | Octanoic acid, 3-methylbutyl ester | 2035-99-6 | C13H26O2 | 31.05 | 1651 |
| 114 |  | Undecanoic acid, methyl ester | 1731-86-8 | C12H24O2 | 32.2 | 1689 |
| 127 |  | methyl phenylacetate | 101-41-7 | C9H10O2 | 33.997 | 1753 |
| 128 |  | Benzoic acid, 2-hydroxy-, methyl ester | 119-36-8 | C8H8O3 | 34.622 | 1774 |
| 129 |  | Dodecanoic acid, methyl ester | 111-82-0 | C13H26O2 | 35.113 | 1792 |
| 130 |  | B-Phenylethyl acetate | 103-45-7 | C10H12O2 | 35.539 | 1808 |
| 131 |  | Benzoic acid, 2-hydroxy-, ethyl ester | 118-61-6 | C9H10O3 | 35.571 | 1809 |
| 135 |  | Dodecanoic acid, ethyl ester | 106-33-2 | C14H28O2 | 36.214 | 1833 |
| 140 |  | Ethyl 3-phenylpropionate | 2021-28-5 | C11H14O2 | 37.423 | 1879 |
| 153 |  | Tetradecanoic acid, methyl ester | 124-10-7 | C15H30O2 | 40.536 | 1999 |
| 161 |  | Pentadecanoic acid, methyl ester | 7132-64-1 | C16H32O2 | 43.07 | 2103 |
| 162 |  | Ethyl (Z)-cinnamate | 4610-69-9 | C11H12O2 | 43.616 | 2126 |
| 170 |  | Hexadecanoic acid, methyl ester | 112-39-0 | C17H34O2 | 45.509 | 2207 |
| 171 |  | n-Hexyl salicylate | 6259-76-3 | C13H18O3 | 45.603 | 2211 |
| 173 |  | 9-Hexadecenoic acid, methyl ester, (Z)- | 1120-25-8 | C17H32O2 | 46.128 | 2235 |
| 174 |  | Hexadecanoic acid, ethyl ester | 628-97-7 | C18H36O2 | 46.351 | 2245 |
| 177 |  | Dimethyl phthalate | 131-11-3 | C10H10O4 | 47.413 | 2293 |
| 178 |  | Heptadecanoic acid, methyl ester | 1731-92-6 | C18H36O2 | 47.832 | 2313 |
| 184 |  | 9-Octadecenoic acid (Z)-, methyl ester | 112-62-9 | C19H36O2 | 50.581 | 2437 |
| 187 |  | 9,12,15-Octadecatrienoic acid, methyl ester, (Z,Z,Z)- | 301-00-8 | C19H32O2 | 53.431 | 2552 |
| 121 | Hydrocarbons | alpha-Farnesene | 502-61-4 | C15H24 | 33.683 | 1741 |
| 33 |  | Dodecane | 112-40-3 | C12H26 | 17.609 | 1237 |
| 46 |  | Tridecane | 629-50-5 | C13H28 | 19.725 | 1298 |
| 109 |  | Heptadecane | 629-78-7 | C17H36 | 31.4 | 1662 |
| 4 | Ketones | 2,3-Pentanedione | 600-14-6 | C5H8O2 | 11.331 | 1048 |
| 10 |  | 3-Penten-2-one | 625-33-2 | C5H8O | 13.622 | 1121 |
| 21 |  | 2-Heptanone | 110-43-0 | C7H14O | 15.404 | 1173 |
| 35 |  | 3-Octanone | 106-68-3 | C8H16O | 17.875 | 1244 |
| 41 |  | Acetoin | 513-86-0 | C4H8O2 | 19.094 | 1280 |
| 45 |  | 1-Octen-3-one | 4312-99-6 | C8H14O | 19.551 | 1293 |
| 49 |  | 2,3-Octanedione | 585-25-1 | C8H14O2 | 20.252 | 1312 |
| 54 |  | 6-Methyl-5-hepten-2-one | 110-93-0 | C8H14O | 20.756 | 1328 |
| 67 |  | 3-Octen-2-one | 1669-44-9 | C8H14O | 23.189 | 1399 |
| 82 |  | 3-Nonen-2-one | 14309-57-0 | C9H16O | 26.546 | 1503 |
| 94 |  | Isophorone | 78-59-1 | C9H14O | 29.244 | 1590 |
| 136 |  | 5,9-Undecadien-2-one, 6,10-dimethyl-, (Z)- | 3879-26-3 | C13H22O | 36.518 | 1845 |
| 160 |  | 2,6-di(t-butyl)-4-hydroxy-4-methyl-2,5-cycloh  exadien-1-one | 10396-80-2 | C15H24O2 | 42.53 | 2081 |
|  |  | exadien-1-one |  |  |  |  |
| 16 | Terpenes | beta-Pinene | 127-91-3 | C10H16 | 14.489 | 1147 |
| 25 |  | dl-Limonene | 138-86-3 | C10H16 | 15.909 | 1188 |
| 32 |  | gamma-Terpinene | 99-85-4 | C10H16 | 17.582 | 1237 |
| 72 | Acids | Acetic acid | 64-19-7 | C2H4O2 | 24.502 | 1440 |
| 100 |  | Butanoic acid | 107-92-6 | C4H8O2 | 30.004 | 1615 |
| 133 |  | Hexanoic acid | 142-62-1 | C6H12O2 | 36.136 | 1830 |
| 145 |  | Heptanoic acid | 111-14-8 | C7H14O2 | 38.952 | 1937 |
| 159 |  | Octanoic acid | 124-07-2 | C8H16O2 | 41.629 | 2044 |
| 175 |  | Decanoic acid | 334-48-5 | C10H20O2 | 46.605 | 2257 |
| 68 | Aldehydes | Hexanal | 66-25-1 | C6H12O | 12.046 | 1072 |
| 69 |  | 2-Pentenal, (E)- | 1576-87-0 | C5H8O | 13.694 | 1123 |
| 6 |  | Heptanal | 111-71-7 | C7H14O | 15.49 | 1176 |
| 29 |  | 2-Hexenal, (E)- | 6728-26-3 | C6H10O | 16.732 | 1212 |
| 42 |  | Octanal | 124-13-0 | C8H16O | 19.135 | 1280 |
| 51 |  | 2-Heptenal, (E)- | 18829-55-5 | C7H12O | 20.408 | 1318 |
| 65 |  | Nonanal | 124-19-6 | C9H18O | 22.741 | 1386 |
| 68 |  | 2-Octenal | 2363-89-5 | C8H14O | 23.961 | 1423 |
| 77 |  | 2,4-Heptadienal,(E,E)- | 4313-03-5 | C7H10O | 25.084 | 1457 |
| 80 |  | Decanal | 112-31-2 | C10H20O | 26.114 | 1489 |
| 83 |  | Benzaldehyde | 100-52-7 | C7H6O | 27.057 | 1519 |
| 86 |  | 2-Nonenal, (E)- | 18829-56-6 | C9H16O | 27.343 | 1528 |
| 92 |  | 2,6-Nonadienal, (E,Z)- | 557-48-2 | C9H14O | 28.932 | 1578 |
| 105 |  | 2-Decenal, (E)- | 3913-81-3 | C10H18O | 30.666 | 1637 |
| 106 |  | Benzeneacetaldehyde | 122-78-1 | C8H8O | 30.69 | 1638 |
| 112 |  | Benzaldehyde, 2-hydroxy- | 90-02-8 | C7H6O2 | 31.82 | 1677 |
| 116 |  | Trans,Trans-Nona-2,4-dienal | 5910-87-2 | C9H14O | 32.355 | 1693 |
| 120 |  | Benzaldehyde, 4-ethyl- | 4748-78-1 | C9H10O | 33.555 | 1737 |
| 123 |  | (E)-2-Undecenal | 53448-07-0 | C11H20O | 33.797 | 1745 |
| 137 |  | (E)-2-Dodecenal | 20407-84-5 | C12H22O | 36.756 | 1854 |
| 157 |  | Benzaldehyde, 4-methoxy- | 123-11-5 | C8H8O2 | 41.121 | 2023 |
| 163 |  | hexadecanal | 629-80-1 | C16H32O | 43.64 | 2127 |
| 57 | Lactones | Cis-Rose Oxide | 876-17-5 | C10H18O | 21.373 | 1346 |
| 101 |  | Butyrolactone | 96-48-0 | C4H6O2 | 30.35 | 1627 |
| 117 |  | 2(3H)-Furanone, 5-ethyldihydro- | 695-06-7 | C6H10O2 | 32.473 | 1699 |
| 149 |  | 2H-Pyran-2-one, tetrahydro-6-propyl- | 698-76-0 | C8H14O2 | 39.678 | 1966 |
| 158 |  | 2(3H)-Furanone, dihydro-5-pentyl- | 104-61-0 | C9H16O2 | 41.2 | 2026 |
| 181 |  | 2(4H)-Benzofuranone, 5,6,7,7a-tetrahydro-4,4,7a-trimethyl- | 15356-74-8 | C11H16O2 | 48.788 | 2356 |
|  |  | 5,6,7,7a-tetrahydro-4,4,7a-trimethyl- |  |  |  |  |
| 183 |  | sigma-Dodecalactone | 713-95-1 | C12H22O2 | 50.321 | 2425 |
| 122 | Naphthalens | Naphthalene | 91-20-3 | C10H8 | 33.719 | 1742 |
| 138 |  | Naphthalene, 2-methyl- | 91-57-6 | C11H10 | 36.811 | 1855 |
| 141 |  | Naphthalene, 1-methyl- | 90-12-0 | C11H10 | 37.785 | 1891 |
| 152 |  | Naphthalene, 1,8-dimethyl- | 569-41-5 | C12H12 | 40.502 | 1998 |
| 154 |  | Naphthalene, 1,6-dimethyl- | 575-43-9 | C12H12 | 40.68 | 2005 |
| 172 |  | Naphthalene, 1,6,7-trimethyl- | 2245-38-7 | C13H14 | 46.022 | 2231 |
| 9 | Furans | 2-Butylfuran | 4466-24-4 | C8H12O | 13.522 | 1117 |
| 30 |  | Furan, 2-pentyl- | 3777-69-3 | C9H14O | 17.007 | 1220 |
| 52 |  | Furan, 2-hexyl- | 3777-70-6 | C10H16O | 20.53 | 1322 |
| 70 |  | 2-Heptyl-furan | 3777-71-7 | C11H18O | 24.11 | 1428 |
| 176 |  | Dibenzofuran | 132-64-9 | C12H8O | 46.952 | 2273 |
| 38 | Sulfur-nitrogen compounds | Thiocyanic acid, methyl ester | 556-64-9 | C2H3NS | 18.652 | 1267 |
| 44 |  | Propane, 1-isothiocyanato- | 628-30-8 | C4H7NS | 19.475 | 1290 |
| 59 |  | Allyl Isothiocyanate | 57-06-7 | C4H5NS | 21.664 | 1354 |
| 74 |  | 1-Butene, 4-isothiocyanato- | 3386-97-8 | C5H7NS | 24.834 | 1450 |
| 148 |  | Benzothiazole | 95-16-9 | C7H5NS | 39.434 | 1956 |
| 5 | Sulfur compounds | Disulfide, dimethyl | 624-92-0 | C2H6S2 | 11.834 | 1065 |
| 15 |  | Methyl ethyl disulfide | 20333-39-5 | C3H8S2 | 14.09 | 1135 |
| 18 |  | Thiophene, 2-ethyl- | 872-55-9 | C6H8S | 15.036 | 1163 |
| 39 |  | Disulfide, methyl 2-propenyl | 2179-58-0 | C4H8S2 | 18.895 | 1275 |
| 63 |  | Trisulfide, dimethyl | 3658-80-8 | C2H6S3 | 22.418 | 1377 |
| 75 |  | Thiophene, 2-pentyl- | 4861-58-9 | C9H14S | 24.985 | 1455 |
| 84 |  | 2-(Methylthio)ethanol | 5271-38-5 | C3H8OS | 27.127 | 1522 |
| 89 |  | Ethyl-3-Methyl Thiopropionate | 13327-56-5 | C6H12O2S | 28.27 | 1558 |
| 95 |  | Trisulfide, methyl 2-propenyl | 34135-85-8 | C4H8S3 | 29.265 | 1589 |
| 108 |  | Disulfide, methyl (methylthio)methyl | 42474-44-2 | C3H8S3 | 31.34 | 1660 |
| 115 |  | 2-Thiophenecarboxaldehyde | 98-03-3 | C5H4OS | 32.208 | 1690 |
| 118 |  | 1-Propanol, 3-(methylthio)- | 505-10-2 | C4H10OS | 32.648 | 1705 |
| 17 | Nitrogen compounds | 2-Butenenitrile | 4786-20-3 | C4H5N | 14.615 | 1150 |
| 20 |  | 3-Butenenitrile | 109-75-1 | C4H5N | 15.35 | 1172 |
| 43 |  | Hexanenitrile | 628-73-9 | C6H11N | 19.473 | 1291 |
| 50 |  | Pyrazine, 2,3-dimethyl- | 5910-89-4 | C6H8N2 | 20.267 | 1314 |
| 58 |  | Hexanenitrile, 5-methyl- | 19424-34-1 | C7H13N | 21.515 | 1351 |
| 90 |  | Pyridine, 2-pentyl- | 2294-76-0 | C10H15N | 28.479 | 1565 |
| 97 |  | Ethanone, 1-(2-pyridinyl)- | 1122-62-9 | C7H7NO | 29.46 | 1597 |
| 143 |  | Benzonitrile, 4-methyl- | 104-85-8 | C8H7N | 38.603 | 1924 |
| 144 |  | Isoquinoline | 119-65-3 | C9H7N | 38.91 | 1936 |
| 185 |  | Indole | 120-72-9 | C8H7N | 50.788 | 2446 |
| 186 |  | Indole, 3-methyl- | 83-34-1 | C9H9N | 51.906 | 2494 |
| 76 | Furfurals | Furfural | 98-01-1 | C5H4O2 | 25.02 | 1456 |
| 91 |  | 5-methyl furfural | 620-02-0 | C6H6O2 | 28.51 | 1566 |
| 104 |  | 5-Ethyl-2-furaldehyde | 23074-10-4 | C7H8O2 | 30.471 | 1631 |
| 146 | Phenols | Creosol | 93-51-6 | C8H10O2 | 39.205 | 1947 |
| 151 |  | Phenol | 108-95-2 | C6H6O | 40.404 | 1994 |
| 155 |  | Phenol, 4-ethyl-2-methoxy- | 2785-89-9 | C9H12O2 | 41.066 | 2021 |
| 165 |  | Eugenol | 97-53-0 | C10H12O2 | 44.371 | 2158 |
| 166 |  | Phenol, 4-ethyl- | 123-07-9 | C8H10O | 44.497 | 2163 |
| 167 |  | 2-Methoxy-4-vinylphenol | 7786-61-0 | C9H10O2 | 45.053 | 2187 |
| 168 |  | Phenol, 2-methyl-5-(1-methylethyl)- | 499-75-2 | C10H14O | 45.344 | 2200 |
| 182 |  | 4-vinylphenol | 2628-17-3 | C8H8O | 49.384 | 2384 |
| 7 | Alcohols | 1-Propanol, 2-methyl- | 78-83-1 | C4H10O | 12.474 | 1086 |
| 8 |  | 1-Butanol, 3-Methyl- | 123-51-3 | C5H12O | 16.229 | 1198 |
| 27 |  | 1,8-Cineole | 470-82-6 | C10H18O | 16.278 | 1199 |
| 34 |  | 1-Pentanol | 71-41-0 | C5H12O | 17.766 | 1241 |
| 48 |  | 2-Heptanol | 543-49-7 | C7H16O | 20.047 | 1307 |
| 56 |  | 1-Hexanol | 111-27-3 | C6H14O | 21.171 | 1341 |
| 62 |  | 3-Hexen-1-ol, (Z)- | 928-96-1 | C6H12O | 22.25 | 1372 |
| 66 |  | 2-Hexen-1-ol, (Z)- | 928-94-9 | C6H12O | 22.919 | 1392 |
| 71 |  | 1-Oocten-3-ol | 53907-72-5 | C8H16O | 24.341 | 1435 |
| 81 |  | 2-Nonanol | 628-99-9 | C9H20O | 26.532 | 1502 |
| 87 |  | Linalool | 78-70-6 | C10H18O | 27.409 | 1530 |
| 96 |  | Terpinen-4-ol | 562-74-3 | C10H18O | 29.339 | 1593 |
| 98 |  | (Z)-5-Octen-1-ol | 64275-73-6 | C9H18O | 29.588 | 1601 |
| 99 |  | 1,2-Propanediol | 57-55-6 | C3H8O2 | 29.698 | 1604 |
| 111 |  | cis-3-Nonenol | 31502-19-9 | C9H18O | 31.654 | 1671 |
| 113 |  | 3-Cyclohexene-1-methanol, .alpha.,.alpha.,4-trimethyl-, (S)- | 10482-56-1 | C10H18O | 32.079 | 1685 |
|  |  | methyl-, (S)- |  |  |  |  |
| 124 |  | 1-Decanol | 112-30-1 | C10H22O | 33.855 | 1748 |
| 125 |  | beta-Citronellol | 106-22-9 | C10H20O | 33.952 | 1751 |
| 134 |  | Nerol | 106-25-2 | C10H18O | 36.16 | 1831 |
| 139 |  | Benzenemethanol | 100-51-6 | C7H8O | 37.103 | 1867 |
| 142 |  | Benzeneethanol | 60-12-8 | C8H10O | 38.073 | 1903 |
| 147 |  | 1-Dodecanol | 112-53-8 | C12H26O | 39.342 | 1953 |
| 156 |  | 1,6,10-Dodecatrien-3-ol, 3,7,11-trimethyl-, (E)- | 40716-66-3 | C15H26O | 41.11 | 2023 |
|  |  | (E)- |  |  |  |  |
| 1644 |  | Ethanol, 2-phenoxy- | 122-99-6 | C8H10O2 | 43.812 | 2134 |
| 179 |  | trans-Farnesol | 106-28-5 | C15H26O | 48.476 | 2342 |
| 110 | Benzene ethers | Benzene, 1-methoxy-4-(2-propenyl)- | 140-67-0 | C10H12O | 31.499 | 1665 |
| 119 |  | Benzene, 1,2-dimethoxy- | 91-16-7 | C8H10O2 | 32.859 | 1712 |
| 126 |  | Anethole | 104-46-1 | C10H12O | 33.976 | 1752 |
| 132 |  | Trans-Anethole | 4180-23-8 | C10H12O | 35.931 | 1822 |
| 2 | Benzene hydrocarbons | Toluene | 108-88-3 | C7H8 | 10.872 | 1033 |
| 12 |  | Ethylbenzene | 100-41-4 | C8H10 | 13.689 | 1123 |
| 14 |  | p-Xylene | 106-42-3 | C8H10 | 13.896 | 1129 |
| 23 |  | o-Xylene | 95-47-6 | C8H10 | 15.453 | 1175 |
| 28 |  | Benzene, propyl- | 103-65-1 | C9H12 | 16.317 | 1200 |
| 36 |  | Styrene | 100-42-5 | C8H8 | 18.06 | 1250 |
| 55 |  | Benzene, 1,2,3-trimethyl- | 526-73-8 | C9H12 | 20.906 | 1333 |
| 61 |  | Benzene, cyclopropyl- | 873-49-4 | C9H10 | 22.103 | 1367 |
| 78 |  | E-1-phenylbutetene | 1005-64-7 | C10H12 | 25.539 | 1472 |
| 150 |  | Biphenyl | 92-52-4 | C12H10 | 40.305 | 1990 |
| 169 |  | Acenaphthylene | 208-96-8 | C12H8 | 45.428 | 2204 |
| 180 |  | Fluorene | 86-73-7 | C13H10 | 48.657 | 2350 |

**Table S2.** The relative abundance of various VFCs in Bashang suancai samples during different stages of fermentation. A, B, C, D and E represented samples collected on 0d, 1d, 3d, 5d, and 7d, respectively.

| Component Name |  | Percentage of relative abundance (%) | | | |
| --- | --- | --- | --- | --- | --- |
|  | A | B | C | D | E |
| Esters | 3.64 | 1.00 | 0.44 | 7.72 | 6.28 |
| Hydrocarbons | 0.13 | 0.05 | 0.05 | 0.14 | 0.12 |
| Ketones | 3.10 | 1.33 | 0.20 | 3.26 | 3.02 |
| Terpenes | 0.25 | 0.03 | 0.02 | 0.55 | 0.45 |
| Acids | 11.13 | 1.27 | 0.40 | 4.71 | 3.67 |
| Aldehydes | 7.90 | 3.42 | 3.21 | 2.48 | 2.48 |
| Lactones | 0.80 | 0.09 | 0.03 | 1.78 | 1.21 |
| Naphthalens | 1.40 | 0.36 | 0.38 | 0.93 | 0.79 |
| Furans | 2.11 | 1.96 | 0.54 | 0.61 | 0.61 |
| Sulfur-nitrogen compounds compounds compounds | 44.88 | 87.05 | 90.67 | 10.45 | 38.19 |
| Sulfur compounds | 1.12 | 0.59 | 0.82 | 0.66 | 0.46 |
| Nitrogen compounds | 3.97 | 0.73 | 1.65 | 8.66 | 5.52 |
| Furfurals | 0.08 | 0.02 | 0.01 | 0.06 | 0.05 |
| Phenols | 1.04 | 0.28 | 0.05 | 1.45 | 0.97 |
| Alcohols | 15.77 | 1.12 | 0.84 | 53.26 | 33.79 |
| Benzene ethers | 0.50 | 0.11 | 0.11 | 0.61 | 0.47 |
| Benzene hydrocarbons | 1.51 | 0.41 | 0.34 | 1.64 | 1.15 |

**Table S3.** Alpha diversity of microbial community during Bashang suancai fermentation. A, B, C, D and E represented samples collected on 0d, 1d, 3d, 5d, and 7d, respectively. Each group had 3 replicates.

| Sample | OTUs | Ace | Chao | Shannon | Simpson | Coverage |
| --- | --- | --- | --- | --- | --- | --- |
| A1 | 232 | 278.69 | 265.00 | 0.68 | 2.61 | 0.9989 |
| A2 | 205 | 257.50 | 252.52 | 0.69 | 2.64 | 0.9990 |
| A3 | 381 | 484.91 | 469.48 | 0.65 | 2.57 | 0.9984 |
| B1 | 215 | 308.06 | 300.02 | 0.70 | 2.23 | 0.9988 |
| B2 | 281 | 554.51 | 430.53 | 0.73 | 2.38 | 0.9979 |
| B3 | 345 | 500.45 | 490.89 | 0.73 | 2.42 | 0.9981 |
| C1 | 353 | 504.17 | 474.64 | 0.77 | 2.68 | 0.9982 |
| C2 | 258 | 344.47 | 330.23 | 0.70 | 2.39 | 0.9981 |
| C3 | 306 | 423.19 | 407.48 | 0.76 | 2.59 | 0.9985 |
| D1 | 219 | 537.15 | 381.30 | 0.74 | 2.42 | 0.9986 |
| D2 | 294 | 432.01 | 378.79 | 0.76 | 2.53 | 0.9985 |
| D3 | 404 | 483.01 | 458.72 | 0.77 | 2.69 | 0.9986 |
| E1 | 292 | 391.02 | 397.21 | 0.68 | 2.23 | 0.9986 |
| E2 | 371 | 487.08 | 481.52 | 0.65 | 2.18 | 0.9984 |
| E3 | 293 | 552.98 | 434.94 | 0.65 | 2.12 | 0.9983 |

**Table S4.** Correlation coefficient between microbial species and VFCs during fermentation.

| Source | Target | Correlation | Weight | *P* value |
| --- | --- | --- | --- | --- |
| Latilactobacillus | Acetic acid | -0.739 | 0.739 | 1.64E-03 |
| Levilactobacillus | Acetic acid | -0.889 | 0.889 | 9.19E-06 |
| Vibrio | Hexanoic acid | -0.718 | 0.718 | 2.58E-03 |
| Latilactobacillus | Heptanoic acid | -0.771 | 0.771 | 7.57E-04 |
| Levilactobacillus | Heptanoic acid | -0.789 | 0.789 | 4.67E-04 |
| Latilactobacillus | Octanoic acid | -0.846 | 0.846 | 6.92E-05 |
| Levilactobacillus | Octanoic acid | -0.871 | 0.871 | 2.32E-05 |
| Weissella | Octanoic acid | -0.886 | 0.886 | 1.12E-05 |
| Vibrio | Decanoic acid | -0.711 | 0.711 | 2.98E-03 |
| Vibrio | 2-Nonanol | -0.757 | 0.757 | 1.08E-03 |
| other | Linalool | -0.718 | 0.718 | 2.58E-03 |
| unclassified_Bacteria | Linalool | -0.868 | 0.868 | 2.75E-05 |
| unclassified_Bacteria | beta-Citronellol | -0.857 | 0.857 | 4.45E-05 |
| Vibrio | Nerol | -0.704 | 0.704 | 3.42E-03 |
| Lactiplantibacillus | 1-Pentanol | 0.743 | 0.743 | 1.51E-03 |
| Latilactobacillus | 2-Heptanol | 0.914 | 0.914 | 1.85E-06 |
| Levilactobacillus | 2-Heptanol | 0.829 | 0.829 | 1.35E-04 |
| Latilactobacillus | Octanal | -0.857 | 0.857 | 4.54E-05 |
| Levilactobacillus | Octanal | -0.950 | 0.950 | 6.22E-08 |
| Weissella | Octanal | -0.778 | 0.778 | 6.41E-04 |
| Latilactobacillus | Nonanal | -0.729 | 0.729 | 2.07E-03 |
| Latilactobacillus | Decanal | -0.746 | 0.746 | 1.39E-03 |
| Levilactobacillus | Decanal | -0.875 | 0.875 | 1.95E-05 |
| Vibrio | 2,6-Nonadienal, (E,Z)- | -0.893 | 0.893 | 7.49E-06 |
| Latilactobacillus | 2-Decenal, (E)- | -0.786 | 0.786 | 5.16E-04 |
| Levilactobacillus | 2-Decenal, (E)- | -0.786 | 0.786 | 5.16E-04 |
| Latilactobacillus | Benzaldehyde, 2-hydroxy- | -0.736 | 0.736 | 1.77E-03 |
| Levilactobacillus | Benzaldehyde, 2-hydroxy- | -0.718 | 0.718 | 2.58E-03 |
| Latilactobacillus | (E)-2-Undecenal | -0.825 | 0.825 | 1.53E-04 |
| Levilactobacillus | (E)-2-Undecenal | -0.818 | 0.818 | 1.95E-04 |
| unclassified_Bacteria | 2-Octenal | 0.707 | 0.707 | 3.20E-03 |
| Vibrio | (E)-2-Dodecenal | 0.746 | 0.746 | 1.39E-03 |
| Vibrio | Benzaldehyde, 4-methoxy- | 0.721 | 0.721 | 2.40E-03 |
| Vibrio | Benzene, propyl- | -0.874 | 0.874 | 2.02E-05 |
| Weissella | Benzene, propyl- | -0.858 | 0.858 | 4.25E-05 |
| Levilactobacillus | Benzene, cyclopropyl- | -0.704 | 0.704 | 3.42E-03 |
| other | Butanoic acid, 2-methyl-, ethyl ester | -0.746 | 0.746 | 1.42E-03 |
| unclassified_Bacteria | Butanoic acid, 2-methyl-, ethyl ester | -0.824 | 0.824 | 1.57E-04 |
| unclassified_Bacteria | Pentanoic acid, ethyl ester | -0.743 | 0.743 | 1.51E-03 |
| other | Hexanoic acid, ethyl ester | -0.779 | 0.779 | 6.27E-04 |
| unclassified_Bacteria | Hexanoic acid, ethyl ester | -0.904 | 0.904 | 3.88E-06 |
| unclassified_Bacteria | Acetic acid, heptyl ester | -0.871 | 0.871 | 2.37E-05 |
| Vibrio | Octanoic acid, ethyl ester | -0.732 | 0.732 | 1.91E-03 |
| other | Undecanoic acid, methyl ester | -0.757 | 0.757 | 1.08E-03 |
| unclassified_Bacteria | Undecanoic acid, methyl ester | -0.739 | 0.739 | 1.64E-03 |
| unclassified_Bacteria | methyl phenylacetate | -0.880 | 0.880 | 1.54E-05 |
| Latilactobacillus | Benzoic acid, 2-hydroxy-, ethyl ester | -0.718 | 0.718 | 2.58E-03 |
| Levilactobacillus | Benzoic acid, 2-hydroxy-, ethyl ester | -0.861 | 0.861 | 3.81E-05 |
| Latilactobacillus | Tetradecanoic acid, methyl ester | -0.707 | 0.707 | 3.20E-03 |
| Levilactobacillus | Tetradecanoic acid, methyl ester | -0.714 | 0.714 | 2.77E-03 |
| Latilactobacillus | 9,12,15-Octadecatrienoic acid, methyl ester, (Z,Z,Z)- | -0.729 | 0.729 | 2.07E-03 |
| Lactiplantibacillus | Butanoic acid, 2-methyl-, ethyl ester | 0.760 | 0.760 | 1.01E-03 |
| Latilactobacillus | Pentanoic acid, ethyl ester | 0.771 | 0.771 | 7.57E-04 |
| Levilactobacillus | Pentanoic acid, ethyl ester | 0.711 | 0.711 | 2.98E-03 |
| Lactiplantibacillus | Hexanoic acid, ethyl ester | 0.704 | 0.704 | 3.42E-03 |
| Lactiplantibacillus | Octanoic acid, methyl ester | 0.711 | 0.711 | 2.98E-03 |
| Lactiplantibacillus | Undecanoic acid, methyl ester | 0.946 | 0.946 | 9.45E-08 |
| Vibrio | Furfural | -0.821 | 0.821 | 1.73E-04 |
| Vibrio | 5-methyl furfural | 0.721 | 0.721 | 2.40E-03 |
| unclassified_Bacteria | 5-Ethyl-2-furaldehyde | 0.793 | 0.793 | 4.22E-04 |
| Vibrio | 3-Penten-2-one | -0.832 | 0.832 | 1.19E-04 |
| Lactiplantibacillus | 3-Octen-2-one | -0.718 | 0.718 | 2.58E-03 |
| Vibrio | 2,3-Pentanedione | 0.850 | 0.850 | 5.96E-05 |
| unclassified_Bacteria | 3-Octen-2-one | 0.782 | 0.782 | 5.70E-04 |
| Latilactobacillus | 3-Nonen-2-one | 0.764 | 0.764 | 9.07E-04 |
| Levilactobacillus | 3-Nonen-2-one | 0.743 | 0.743 | 1.51E-03 |
| other | Cis-Rose Oxide | -0.733 | 0.733 | 1.86E-03 |
| unclassified_Bacteria | Cis-Rose Oxide | -0.905 | 0.905 | 3.64E-06 |
| Vibrio | 2(3H)-Furanone, 5-ethyldihydro- | -0.739 | 0.739 | 1.64E-03 |
| unclassified_Bacteria | 2H-Pyran-2-one, tetrahydro-6-propyl- | -0.856 | 0.856 | 4.64E-05 |
| Latilactobacillus | Naphthalene, 1-methyl- | -0.714 | 0.714 | 2.77E-03 |
| Levilactobacillus | Naphthalene, 1-methyl- | -0.721 | 0.721 | 2.40E-03 |
| unclassified_Bacteria | Naphthalene, 2-methyl- | 0.889 | 0.889 | 9.19E-06 |
| unclassified_Bacteria | Naphthalene, 1-methyl- | 0.807 | 0.807 | 2.75E-04 |
| Vibrio | Hexanenitrile | -0.832 | 0.832 | 1.19E-04 |
| other | Pyrazine, 2,3-dimethyl- | -0.739 | 0.739 | 1.64E-03 |
| unclassified_Bacteria | Pyrazine, 2,3-dimethyl- | -0.846 | 0.846 | 6.92E-05 |
| unclassified_Bacteria | Ethanone, 1-(2-pyridinyl)- | -0.721 | 0.721 | 2.40E-03 |
| Latilactobacillus | Benzonitrile, 4-methyl- | 0.739 | 0.739 | 1.64E-03 |
| Levilactobacillus | Benzonitrile, 4-methyl- | 0.786 | 0.786 | 5.16E-04 |
| other | Isoquinoline | 0.711 | 0.711 | 2.98E-03 |
| unclassified_Bacteria | Isoquinoline | 0.821 | 0.821 | 1.73E-04 |
| Latilactobacillus | Furan, 2-hexyl- | -0.706 | 0.706 | 3.27E-03 |
| Levilactobacillus | Furan, 2-hexyl- | -0.795 | 0.795 | 3.93E-04 |
| Vibrio | 2-Heptyl-furan | 0.875 | 0.875 | 2.00E-05 |
| other | Creosol | -0.754 | 0.754 | 1.18E-03 |
| unclassified_Bacteria | Creosol | -0.875 | 0.875 | 1.95E-05 |
| Latilactobacillus | Phenol | -0.704 | 0.704 | 3.42E-03 |
| Levilactobacillus | Phenol | -0.807 | 0.807 | 2.75E-04 |
| Latilactobacillus | Phenol, 4-ethyl-2-methoxy- | -0.743 | 0.743 | 1.51E-03 |
| other | Phenol, 4-ethyl-2-methoxy- | 0.736 | 0.736 | 1.77E-03 |
| unclassified_Bacteria | Phenol, 4-ethyl-2-methoxy- | 0.832 | 0.832 | 1.19E-04 |
| Levilactobacillus | Disulfide, dimethyl | -0.743 | 0.743 | 1.51E-03 |
| Latilactobacillus | Methyl ethyl disulfide | 0.789 | 0.789 | 4.67E-04 |
| Levilactobacillus | Methyl ethyl disulfide | 0.779 | 0.779 | 6.27E-04 |
| other | Thiophene, 2-ethyl- | 0.761 | 0.761 | 9.91E-04 |
| unclassified_Bacteria | Thiophene, 2-ethyl- | 0.868 | 0.868 | 2.75E-05 |
| unclassified_Bacteria | Trisulfide, methyl 2-propenyl | 0.779 | 0.779 | 6.27E-04 |
| Vibrio | Disulfide, methyl (methylthio)methyl | 0.736 | 0.736 | 1.77E-03 |
| unclassified_Bacteria | 2-Thiophenecarboxaldehyde | 0.829 | 0.829 | 1.35E-04 |
| Vibrio | Propane, 1-isothiocyanato- | -0.763 | 0.763 | 9.26E-04 |
| Latilactobacillus | Benzothiazole | -0.725 | 0.725 | 2.23E-03 |
| Levilactobacillus | Benzothiazole | -0.732 | 0.732 | 1.91E-03 |
| Weissella | Benzothiazole | -0.779 | 0.779 | 6.27E-04 |
| other | gamma-Terpinene | -0.775 | 0.775 | 6.90E-04 |
| unclassified_Bacteria | gamma-Terpinene | -0.854 | 0.854 | 5.17E-05 |
